# PMEPA1 attenuates myocardial ischemia-reperfusion injury via interaction with NEDD4 and involvement of the TGF-β/SMAD pathway

**DOI:** 10.3389/fphar.2026.1881951

**Published:** 2026-10-01

**Authors:** Weilong Zhong, Yanan Li, Baoze Qu

**Affiliations:** 1 Department of Cardiology, The First Affiliated Hospital of Jinzhou Medical University, Jinzhou, China; 2 Scientific Research Center, Jinzhou Medical University, Jinzhou, China; 3 Department of Pediatrics, Jinzhou Maternal and Child Hospital, Jinzhou, China

**Keywords:** machine learning, myocardial ischemia-reperfusion injury, Nedd4, PMEPA1, protein-protein interaction

## Abstract

**Introduction:**

Myocardial ischemia-reperfusion injury (MIRI) remains a significant challenge in cardiovascular medicine, and its molecular mechanisms are not yet fully elucidated.

**Methods:**

In the present study, integrated transcriptomic analysis combined with machine learning identified PMEPA1 as a key gene consistently downregulated in both rat models of MIRI and H9c2 cardiomyocytes subjected to ischemia –reperfusion injury. A series of *in vivo* and *in vitro* experiments, including gene overexpression and knockdown, cell viability and proliferation assays, flow cytometry, Western blot, co-immunoprecipitation, and immunofluorescence, were performed to investigate the role and underlying mechanism of PMEPA1 in MIRI.

**Results:**

Overexpression of PMEPA1 enhanced cardiomyocyte proliferation, reduced apoptosis, and inhibited activation of the TGF-β/SMAD signaling pathway. Further investigation revealed a direct interaction between PMEPA1 and the E3 ubiquitin ligase NEDD4. Both NEDD4 overexpression and PMEPA1 knockdown aggravated cardiomyocyte injury and increased activation of the TGF-β/SMAD pathway. Notably, the detrimental effects of NEDD4 overexpression were attenuated in the absence of PMEPA1, suggesting a PMEPA1-dependent mechanism.

**Discussion:**

These findings highlight a novel NEDD4–PMEPA1 axis in the regulation of TGF-β/SMAD signaling during myocardial injury and provide new insights into potential molecular targets for the treatment of cardiovascular disease.

## Introduction

Ischemic heart disease remains a leading cause of mortality worldwide and continues to pose a significant public health challenge (He et al., 2020). Timely reperfusion therapy is currently the cornerstone of treatment for restoring coronary blood flow to ischemic myocardium, which can partially mitigate tissue injury and improve clinical outcomes (Hausenloy and Yellon, 2016). However, the abrupt restoration of blood flow also subjects previously ischemic cardiomyocytes to sudden reoxygenation, triggering a cascade of complex pathological events. This sudden reoxygenation often results in secondary injury, exacerbating myocardial necrosis and apoptosis—a phenomenon known as myocardial ischemia/reperfusion (I/R) injury (Chang et al., 2021; Sun et al., 2022; Zhang et al., 2024). Consequently, a comprehensive understanding of the molecular mechanisms underlying myocardial I/R injury, as well as the identification of novel therapeutic targets, is essential for reducing mortality and improving prognosis in patients with ischemic heart disease.

PMEPA1 was originally identified as an androgen-inducible gene in prostate cancer, where it was subsequently shown to play a critical role by modulating the TGF-β signaling pathway, particularly in the context of bone metastasis (Fournier et al., 2015). Beyond prostate cancer, PMEPA1 has been implicated in the progression of various other solid tumors, including renal cell carcinoma (Rae et al., 2001), lung cancer (Vo et al., 2014), and colorectal cancer, primarily through its regulation of TGF-β signaling and its promotion of epithelial-mesenchymal transition (EMT) (Zhang et al., 2019). The biological functions of PMEPA1 are largely attributed to its capacity to regulate the TGF-β signaling network, which is not only pivotal for tumorigenesis but also recognized as a key mediator in the pathogenesis of cardiac fibrosis and heart failure (Ko et al., 2022; Yao et al., 2020). There is growing interest in the idea that molecules associated with cancer may also influence the development of cardiovascular diseases. Many of these effects appear to involve mechanisms similar to those found in oncology. This area has attracted attention in both basic science and clinical research.

NEDD4, an E3 ubiquitin ligase, is well established for its role in ubiquitin-dependent, proteasome-mediated protein degradation and is involved in a wide range of physiological and pathological processes (Wang et al., 2020). Previous studies have demonstrated that PMEPA1 interacts with members of the NEDD4 family to regulate various cellular functions, including vesicular trafficking, cell growth, and protein turnover (Hirata et al., 2021). In prostate cancer, PMEPA1 promotes the recruitment of NEDD4, leading to the ubiquitination and subsequent degradation of the androgen receptor (AR). This process results in decreased AR protein levels and reduced transcriptional activity (Li et al., 2008). Collectively, these findings underscore the regulatory importance of the PMEPA1–NEDD4 interaction in cellular signaling.

Nevertheless, the expression dynamics and biological significance of PMEPA1, as well as its regulatory relationship with NEDD4 in myocardial I/R injury, remain largely unexplored. In this work, we employed bioinformatics analysis together with *in vivo* and *in vitro* experiments to assess the role of PMEPA1 in MIRI and to preliminarily investigate its association with NEDD4. The present findings may help to clarify the molecular networks involved in myocardial injury and could inform future studies aimed at therapeutic development.

## Methods

### Raw data acquisition and preprocessing

RNA-seq data were obtained from the GSE127853 (n = 6) and GSE194388 (n = 15) datasets. The datasets were integrated, and batch effects were corrected using the ComBat algorithm in the “sva” package. Principal component analysis (PCA) was performed to visualize the distribution of samples. All bulk RNA sequencing data were log2-transformed prior to analysis.

### RNA-seq data analysis

Differentially expressed genes (DEGs) were identified using the limma package, with selection thresholds set at |log2 FC|>1 and p < 0.05. Functional enrichment analyses, including Gene Ontology (GO) and Kyoto Encyclopedia of Genes and Genomes (KEGG), were performed using the clusterProfiler package.

Feature genes were screened using LASSO logistic regression, SVM-RFE, and random forest (RF). For LASSO, six-fold cross-validation was used and genes with non-zero coefficients at lambda. min were retained. For SVM-RFE, 10-fold cross-validation was used to identify the feature set with the lowest classification error. For RF, 500 trees were initially constructed, and the top 20 genes ranked by MeanDecreaseGini were selected. Genes shared by all three methods were considered key candidate genes and were further examined experimentally.

### Rat model of myocardial ischemia-reperfusion injury (MIRI)

Adult male Sprague-Dawley rats (220–230 g; n = 12) were obtained from Liaoning Changsheng Biotechnology Co., Ltd. (Liaoning, China). The animals were housed under controlled conditions (25 °C ± 1 °C, 65% ± 5% humidity, 12-h light/dark cycle) with *ad libitum* access to food and water. All experimental procedures were approved by the Animal Ethics Committee of Jinzhou Medical University (approval number: 2025104). Prior to the experiments, rats were acclimatized for at least 1 week.

The animals were randomly assigned to either the sham group or the MIRI group (n = 6 per group). Anesthesia was induced by intraperitoneal injection of sodium pentobarbital (90 mg/kg). Myocardial ischemia was achieved by placing a slipknot around the left anterior descending (LAD) coronary artery just below the left atrial appendage. The artery was occluded for 45 min, after which the ligature was released to allow reperfusion for 24 h. In the sham group, the suture was passed under the LAD without tightening, and all other procedures were performed identically to the MIRI group.

### Histochemical or immunohistochemical staining

Tissue samples were fixed in formalin, dehydrated through a graded ethanol series, and embedded in paraffin. Paraffin blocks were sectioned at a thickness of 4 μm. For histological assessment, sections were stained with hematoxylin and eosin (H&E). For immunohistochemical (IHC) analysis, antigen retrieval and blocking were performed prior to incubation with primary antibodies, following established protocols. Stained slides were examined and imaged using a light microscope (Olympus, Tokyo, Japan).

### ELISA

Serum concentrations of the myocardial injury markers cTn-I and CK-MB were quantified using commercially available enzyme-linked immunosorbent assay (ELISA) kits (MEIMIAN, China), strictly adhering to the manufacturer’s instructions. Absorbance was measured with a microplate reader, and results were analyzed accordingly.

### TUNEL assay

Myocardial tissues were fixed in phosphate-buffered paraformaldehyde, dehydrated in a graded ethanol series, cleared with xylene, and embedded in paraffin. Sections were cut at 5 μm thickness. TUNEL staining was carried out using a commercial kit (Roche, Basel, Switzerland), following the manufacturer’s guidelines.

### RT-PCR

Total RNA was extracted from cells using TRIzol reagent (Invitrogen, Thermo Fisher Scientific, United States of America) following the manufacturer’s protocol. cDNA was synthesized with the PrimeScript™ RT Reagent Kit (Takara, Shiga, Japan). Quantitative PCR was carried out using ChamQ Universal SYBR qPCR Master Mix (Vazyme, China) in accordance with the recommended procedure. Relative gene expression was determined using the 2^^−ΔΔCt^ method. Primer sequences used in this study are provided in Supplementary Table S1.

### Western blot

Proteins were extracted from cultured cells using RIPA lysis buffer, and concentrations were determined prior to separation by SDS-PAGE. Following electrophoresis, proteins were transferred to PVDF membranes and blocked with 5% non-fat milk at room temperature for 2 h. Membranes were then incubated overnight at 4 °C with primary antibodies, followed by incubation with the appropriate secondary antibodies for 2 h at room temperature. Protein bands were visualized using an enhanced chemiluminescence (ECL) kit and imaged with a Syngene imaging system (Syngene, Cambridge, United Kingdom). GAPDH was used as a loading control. Detailed information regarding the antibodies is provided in Supplementary Table S2.

### Cell culture and treatment protocol

The H9c2 rat cardiomyoblast cell line was obtained from the Cell Bank of the Chinese Academy of Sciences (Shanghai, China). An *in vitro* model of I/R injury was established using oxygen-glucose deprivation and subsequent reoxygenation (OGD/R). For OGD, the culture medium was replaced with glucose-free DMEM, and cells were incubated for 6 h in a hypoxic chamber (95% N_2_, 5% CO_2_). Reoxygenation was initiated by replacing the medium with glucose-containing DMEM and returning the cells to normoxic conditions (95% air, 5% CO_2_). Control cells were maintained under normoxic conditions throughout the experiment.

### Cell transfection

After 2 days of culture, H9c2 cells were transfected with either pcDNA3.1 or pcDNA3.1-PMEPA1 plasmids using Lipofectamine 3000 (Invitrogen, United States of America) according to the manufacturer’s instructions. For gene silencing, lentiviral vectors carrying shRNA targeting *PMEPA1*, as well as corresponding control vectors, were obtained from GeneChem (Shanghai, China). The sequences of the shRNA oligonucleotides are listed in Supplementary Table S3.

### CCK-8 assay

Cells were seeded into 96-well plates at a density of 2 × 10^3^ cells per well. Cell proliferation was evaluated at 0 and 24 h using the Cell Counting Kit-8 (CCK-8, Beyotime, Shanghai, China) in accordance with the manufacturer’s instructions. Absorbance at 450 nm was measured with a microplate reader to quantify cell viability.

### Edu staining

Cells were plated in 96-well plates and incubated with EdU reagent (Ribobio, Guangzhou, China). After staining, EdU-positive cells were visualized and imaged using a confocal microscope.

### Apoptosis assay

Both adherent and floating cells were collected, washed with PBS and binding buffer, and resuspended at an appropriate density. Cells were stained with Annexin V-APC for 15 min in the dark, followed by PI staining. Samples were immediately analyzed by flow cytometry.

### CO-IP

Cells were washed with ice-cold PBS and lysed in ice-cold IP lysis buffer. After centrifugation, protein concentrations were determined using the BCA assay. Equal amounts of total protein (1.0–1.2 mg) were incubated with the appropriate primary antibody overnight at 4 °C, followed by the addition of protein A/G agarose beads and further incubation for 1–2 h at 4 °C. Immunocomplexes were washed with lysis buffer and eluted in loading buffer at 95 °C–100 °C. Samples were then separated by SDS-PAGE and transferred to PVDF membranes. Membranes were blocked with 5% non-fat milk, incubated with primary antibodies overnight at 4 °C, and then with secondary antibodies at room temperature. Protein bands were detected by chemiluminescence and analyzed.

### Immunofluorescence staining

Cells were cultured on sterilized coverslips in 24-well plates, fixed with 4% paraformaldehyde. After blocking with 4% bovine serum albumin (BSA), cells were sequentially incubated with primary antibodies followed by fluorescently labeled secondary antibodies. Nuclei were counterstained with Hoechst dye. Coverslips were then mounted, and fluorescence images were captured using a fluorescence microscope.

### Statistical analysis

Statistical analyses were performed using SPSS 25.0 and R version 4.2.2. Data are presented as mean ± SD. Cell experiments were repeated at least three times independently. For two-group comparisons, an independent-samples t-test was used for normally distributed data, while the Wilcoxon rank-sum test was used for non-normally distributed data. One-way ANOVA was used for comparisons among multiple groups. Six rats were included in each group for the animal experiments. A p value <0.05 was considered statistically significant.

## Results

### Identification and functional enrichment analysis of DEGs

After correction for batch effects, PCA showed substantially improved clustering of samples across the two datasets (Figures 1A,B). The integrated analysis included 13 myocardial infarction samples and 8 normal controls, resulting in the identification of 334 DEGs (Figure 1C; Supplementary Table S4).

**FIGURE 1:**
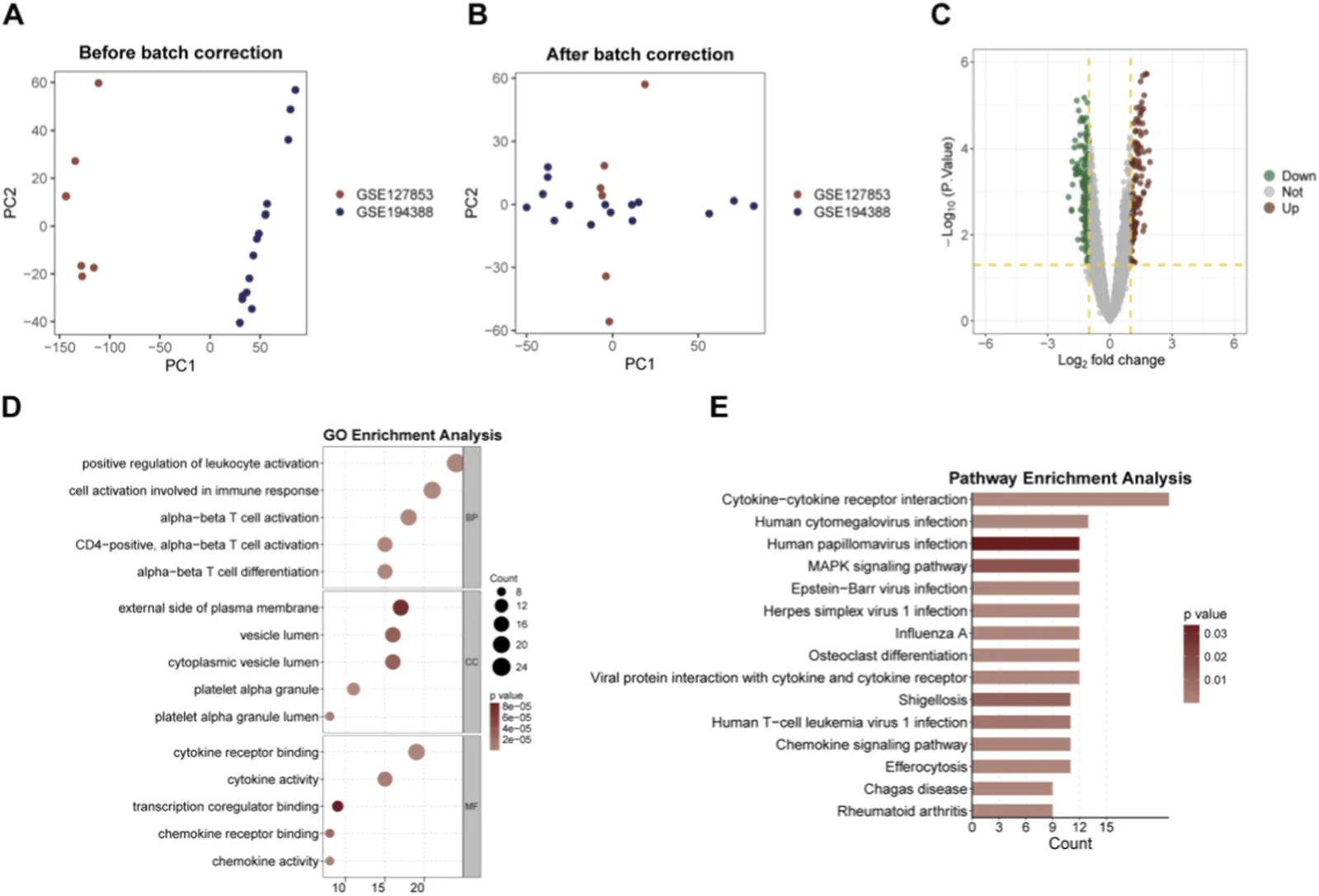
Identification and Functional Enrichment Analysis of DEGs. **(A)** Samples from different databases before batch effect removal; **(B)** After batch effect removal, samples from different databases are more evenly distributed; **(C)** Volcano plot of DEGs; **(D)** GO enrichment analysis of DEGs; **(E)** KEGG pathway enrichment analysis of DEGs.

GO enrichment analysis revealed that, in the biological process (BP) category, DEGs were predominantly involved in immune-related pathways such as leukocyte activation, immune response, and T cell function. In the cellular component (CC) and molecular function (MF) categories, DEGs were mainly associated with cell membranes, vesicles, and cytokine receptor binding (Figure 1D).

KEGG pathway analysis further indicated that DEGs were enriched in pathways related to inflammation, signal transduction, and viral infection, including cytokine–cytokine receptor interactions, the MAPK signaling pathway, and several virus-associated pathways (Figure 1E).

### Identification of hub genes using machine learning algorithms

The LASSO regression algorithm identified 11 candidate hub genes (Figure 2A). SVM analysis selected 18 genes (Figure 2B), while the RF algorithm selected 20 genes (Figure 2C). Cross-comparison of these gene sets revealed two overlapping genes—*PMEPA1* and *PPM1B* (Figure 2D). Both genes showed consistent downregulation in myocardial infarction samples compared to controls (Figures 2E,F).

**FIGURE 2:**
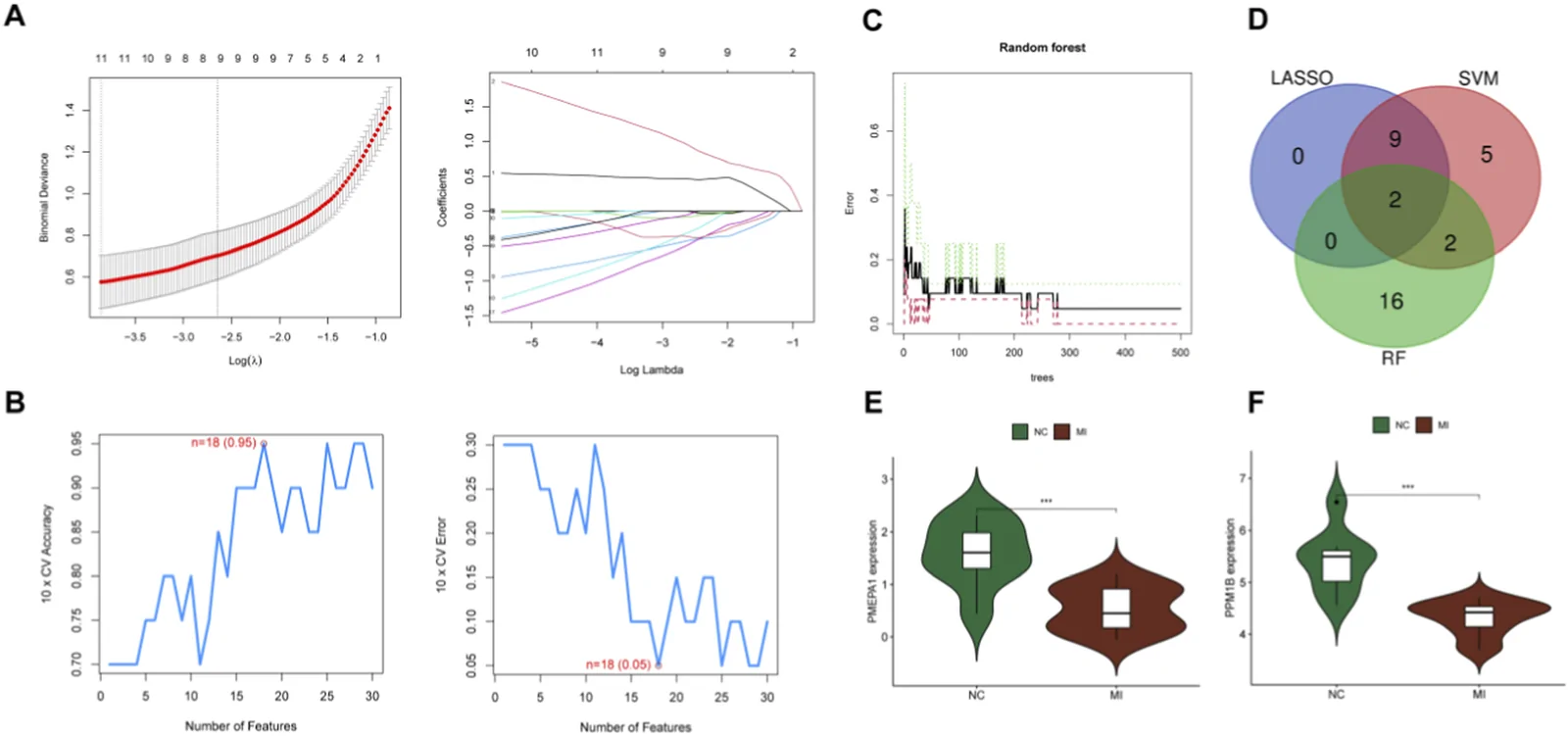
Identification of hub genes using machine learning algorithms. **(A)** The least absolute shrinkage and selection operator (LASSO) logistic regression algorithm; **(B)** RF algorithm; and **(C)** SVM-RFE algorithm were each applied to identify hub genes; **(D)** The Venn diagram illustrates the overlap of hub genes identified by these three algorithms; **(E,F)** Expression levels of *PMEPA1* **(E)** and *PPM1B* **(F)** in NC and disease samples.

### The role of PMEPA1 in regulating the TGF-β/SMAD pathway in MIRI rat model

A rat model of MIRI was generated by ligating the LAD coronary artery (Figure 3A). In the sham group, HE staining revealed normal myocardial architecture. In contrast, the MIRI group showed marked disruption of myocardial fibers, increased infiltration of inflammatory cells, and signs of myocardial edema (Figure 3B). Biochemical assays demonstrated that CK-MB activity and cTnT concentrations were significantly higher in the MIRI group than in the sham group, while serum levels of LDH, CK-MB, and cTnT remained low in the sham animals (Figure 3C). TUNEL staining detected very few apoptotic cells in the sham group, but a marked increase in TUNEL-positive cells was observed in the MIRI group (Figure 3D).

**FIGURE 3:**
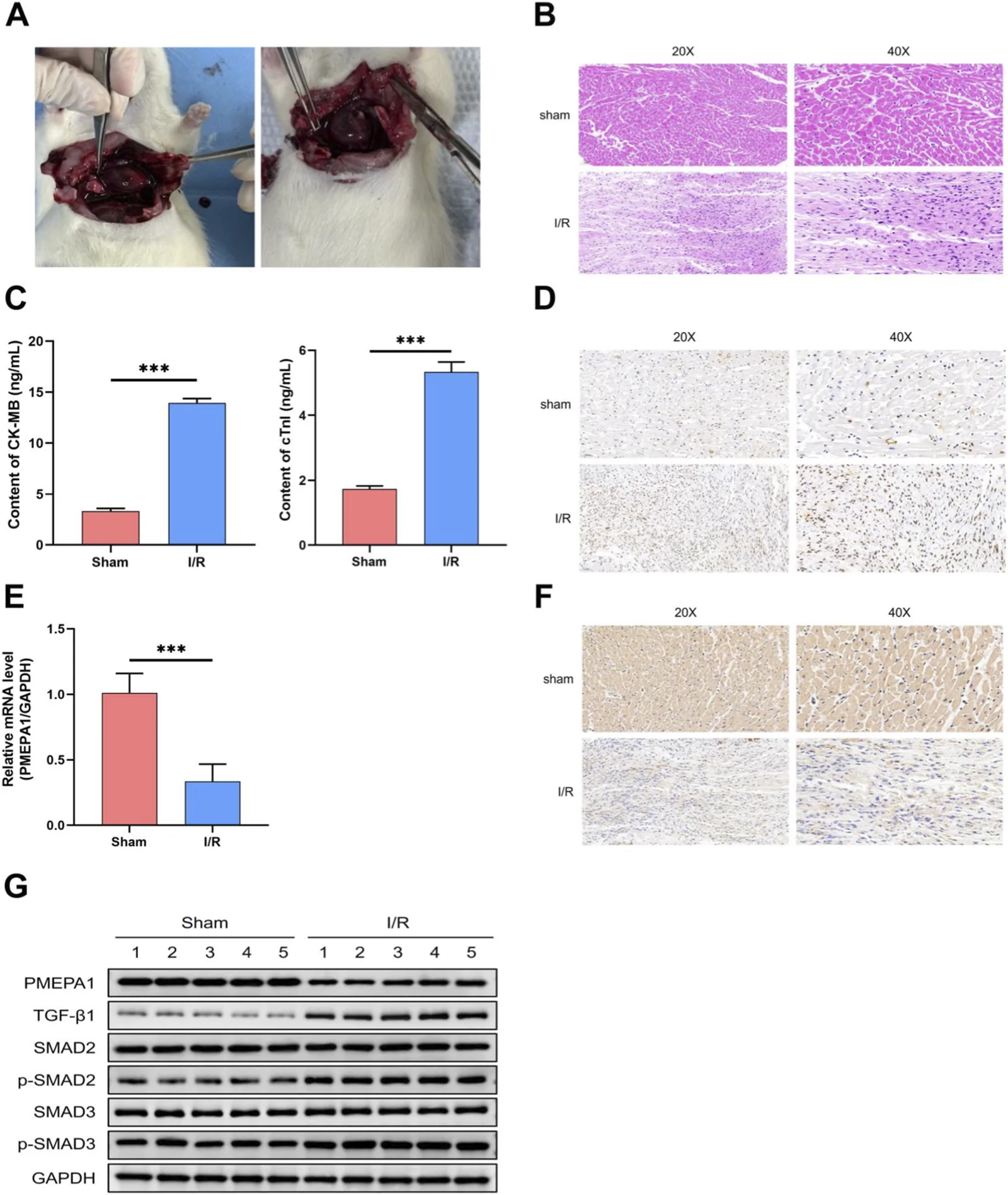
The Role of PMEPA1 in Regulating the TGF-β/SMAD Pathway in a Rat Model of MIRI. **(A)** Establishment of the rat MIRI model; **(B)** Histopathological evaluation of myocardial tissue by H&E staining; **(C)** Assessment of serum LDH and CK-MB activity in rats; **(D)** Detection of cardiomyocyte apoptosis using the TUNEL assay; **(E)** Analysis of *PMEPA1* mRNA expression levels by qRT-PCR; **(F)** Immunohistochemical detection of PMEPA1 expression in myocardial tissue; **(G)** Western blot analysis of PMEPA1 and TGF-β pathway-related proteins in myocardial tissue.

qRT-PCR analysis revealed that PMEPA1 expression in myocardial tissue was significantly reduced in the MIRI group compared with the sham group (Figure 3E). Immunohistochemical staining showed that PMEPA1 was primarily localized to the cytoplasm of cardiomyocytes, and its expression was clearly decreased following I/R injury (Figure 3F). Consistent with these findings, Western blot analysis confirmed lower levels of PMEPA1 protein in the MIRI group. This reduction was accompanied by increased levels of TGF-β1, p-SMAD2, and p- SMAD3, while total SMAD2 and SMAD3 protein levels remained unchanged (Figure 3G).

### PMEPA1 overexpression attenuates OGD/R-induced cellular injury and TGF-β pathway activation

We first assessed the impact of OGD/R on H9c2 cell viability. Cell viability decreased significantly following 6 h of hypoxia and either 4 or 24 h of reoxygenation, compared with untreated controls (Figures 4A,B). As a significant reduction in cell viability was already observed after 4 h of reoxygenation, the OGD-6h/R-4h condition was selected for the subsequent functional experiments. qRT-PCR analysis confirmed that *PMEPA1* mRNA levels were markedly downregulated in cells subjected to OGD/R conditions (Figure 4C). At the protein level, PMEPA1 expression was reduced, while TGF-β1, p-SMAD2, and p-SMAD3 levels were increased; total SMAD2 and SMAD3 levels remained unchanged (Figure 4D).

**FIGURE 4:**
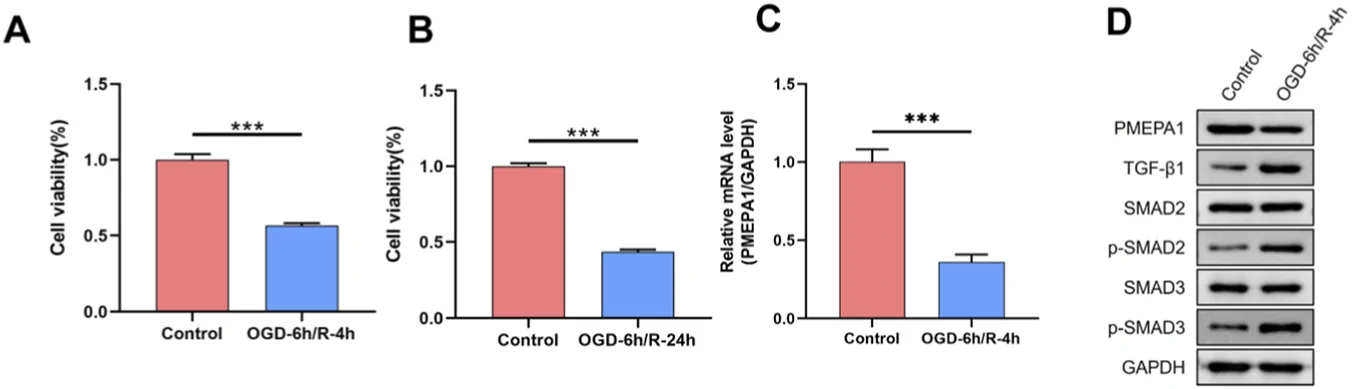
PMEPA1 expression is downregulated in the OGD/R cell model. **(A,B)** Cell viability of H9c2 cells measured by CCK8 assay following 6 h of hypoxia and 4 h **(A)** or 24 h **(B)** of reoxygenation; **(C)** qRT-PCRanalysis of *PMEPA1* mRNA expression in the OGD/R cell model; **(D)** Western blot analysis of PMEPA1 protein expression in the OGD/R cell model. Data are from three independent biological replicates (n = 3).

To investigate the effects of PMEPA1 overexpression, we transfected OGD/R-treated H9c2 cells with pcDNA3.1-PMEPA1. Successful overexpression was validated by both qRT-PCR and Western blot, which showed a significant increase in PMEPA1 mRNA and protein in the OGD-6h/R-4h + PMEPA1 group compared to the negative control (Figures 5A,B).

**FIGURE 5:**
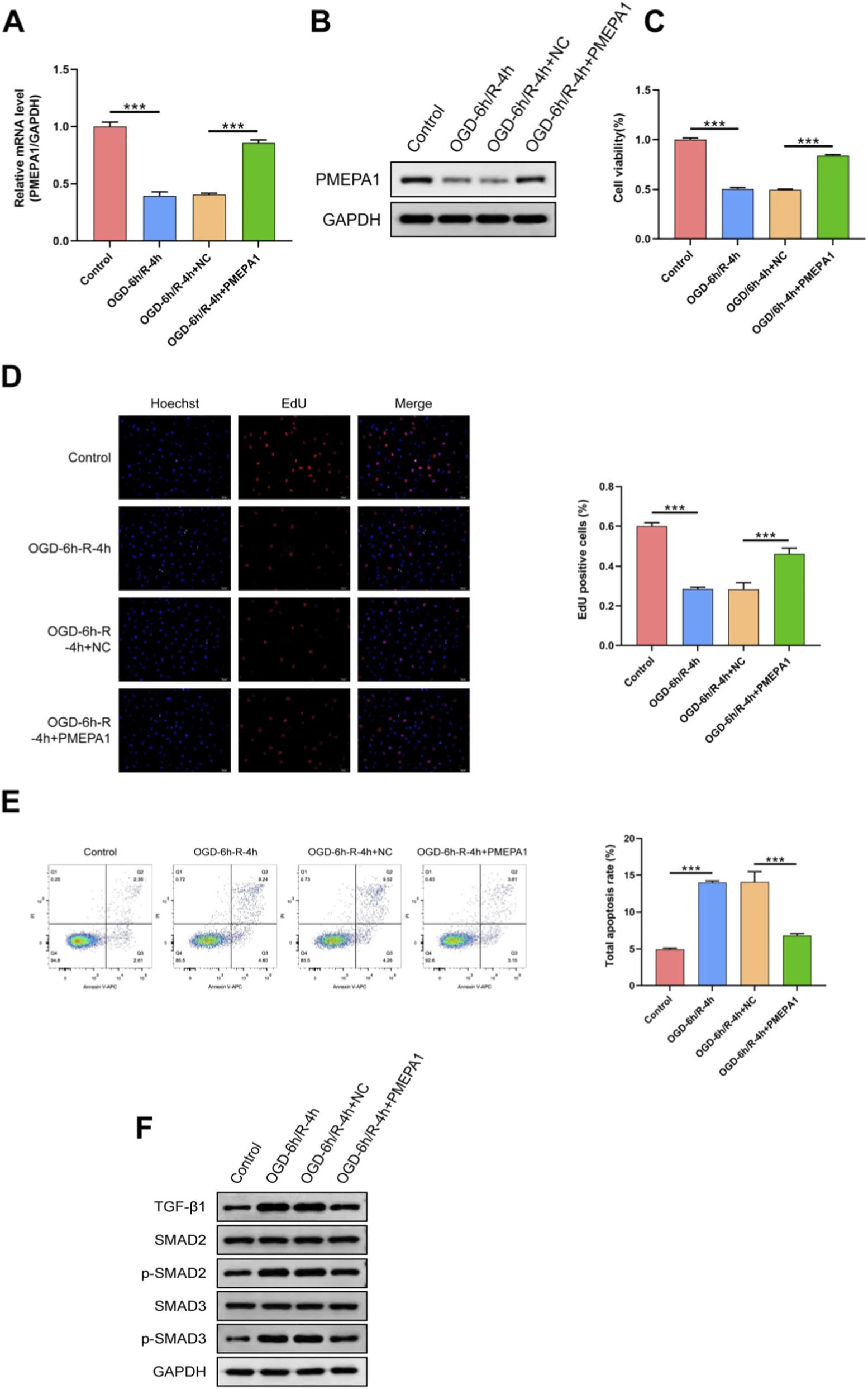
Effects of PMEPA1 overexpression on cellular function and TGF-β signaling in cardiomyocytes. **(A,B)** PMEPA1 overexpression efficiency validated by qRT-PCR **(A)** and Western blot analysis **(B)**; **(C)** Cell viability assessed by CCK8 assay; **(D)** Cell proliferation evaluated by EdU assay; **(E)** Apoptosis determined by flow cytometry; **(F)** Western blot analysis of TGF-β1, p-SMAD2, SMAD2, p-SMAD3, and SMAD3 protein expression. Data are from three independent biological replicates (n = 3).

Next, we evaluated whether PMEPA1 could influence cell survival and proliferation after OGD/R-induced injury. CCK-8 and EdU assays demonstrated that OGD-6h/R-4h exposure significantly impaired cell viability and proliferation, effects that were substantially reversed by PMEPA1 overexpression (Figures 5C,D). Flow cytometry revealed increased apoptosis following OGD-6h/R-4h treatment, whereas overexpression of PMEPA1 significantly reduced the apoptotic rate (Figure 5E). Further analysis indicated that OGD-6h/R-4h upregulated TGF-β1, p-SMAD2, and p-SMAD3 protein levels, but these increases were partially reversed by PMEPA1 overexpression (Figure 5F).

### Interaction between PMEPA1 and NEDD4

To further elucidate the role of PMEPA1 in modulating the TGF-β signaling pathway during myocardial ischemia–reperfusion, potential protein–protein interactions involving PMEPA1 were first predicted using the STRING database. This analysis identified NEDD4, an E3 ubiquitin ligase, as a likely interacting partner of PMEPA1, consistent with previous reports (Hirata et al., 2021; Li et al., 2008; Zhu et al., 2023). As a prominent member of the ubiquitin ligase family, NEDD4 is known to mediate the ubiquitination of various substrate proteins and plays a critical role in post-translational regulation (Boase and Kumar, 2015) (Figure 6A).

**FIGURE 6:**
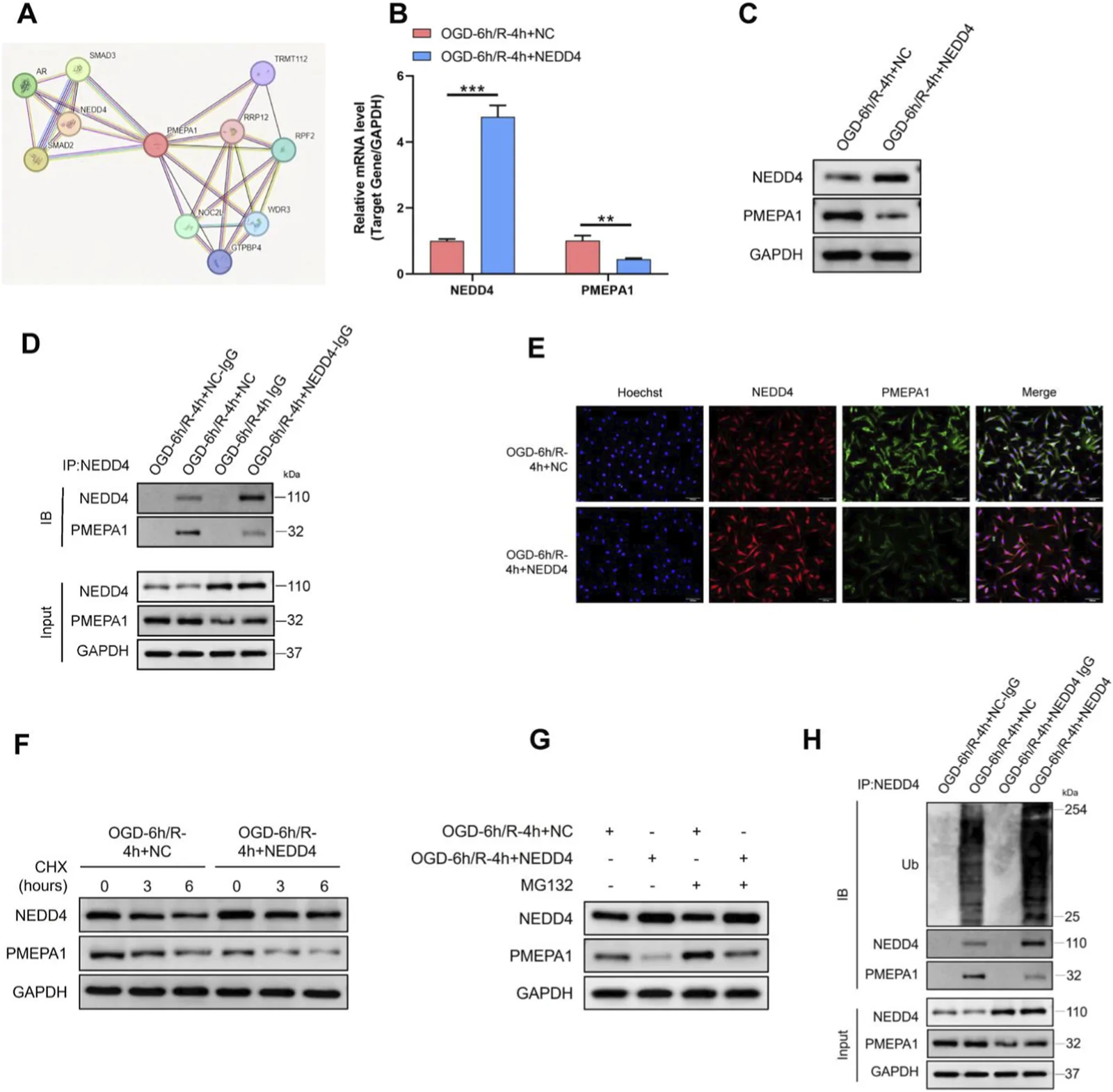
NEDD4 interacts with PMEPA1 and regulates its protein stability **(A)** STRING database prediction of potential PMEPA1-interacting proteins; **(B,C)** qRT-PCR **(B)** and Western blot **(C)** validation of NEDD4 and PMEPA1 expression; **(D)** Representative Western blot images of Co-IP showing the interaction between NEDD4 and PMEPA1; **(E)** Immunofluorescence analysis of the subcellular colocalization of NEDD4 and PMEPA1. **(F)** CHX chase assay examining PMEPA1 protein stability following NEDD4 overexpression; **(G)** Western blot analysis of PMEPA1 expression following MG132 treatment; **(H)** Ubiquitination assay following NEDD4 overexpression. Data are from three independent biological replicates (n = 3).

The impact of NEDD4 overexpression on PMEPA1 expression in cardiomyocytes under OGD/R conditions was next examined. qRT-PCR analysis demonstrated a significant increase in NEDD4 mRNA levels in the OGD-6h/R-4h + NEDD4 group compared to the OGD-6h/R-4h + NC group, while PMEPA1 mRNA expression was significantly decreased (Figure 6B). Western blot analysis corroborated these findings, showing elevated NEDD4 protein and reduced PMEPA1 protein in response to NEDD4 overexpression (Figure 6C).

To determine whether PMEPA1 and NEDD4 physically interact, Co-IP assays were performed. In the OGD-6h/R-4h + NC group, PMEPA1 protein was detected in immunoprecipitates obtained with a NEDD4 antibody, and the strength of this interaction was markedly increased in the OGD-6h/R-4h + NEDD4 group (Figure 6D). Immunofluorescence analysis further demonstrated enhanced cytoplasmic colocalization of NEDD4 and PMEPA1 in cells overexpressing NEDD4 (Figure 6E). CHX chase assays showed that PMEPA1 was degraded more rapidly following NEDD4 overexpression, indicating reduced PMEPA1 protein stability (Figure 6F). This reduction was partially reversed by MG132 treatment, suggesting that proteasomal degradation was involved (Figure 6G). Consistently, stronger ubiquitination-associated signals were observed after NEDD4 overexpression (Figure 6H).

### PMEPA1 overexpression attenuates NEDD4-induced cardiomyocyte injury under OGD/R

PMEPA1 was overexpressed in NEDD4-overexpressing H9c2 cells under OGD/R conditions. qRT-PCR showed increased NEDD4 expression after NEDD4 overexpression, while PMEPA1 overexpression did not markedly alter NEDD4 levels (Figure 7A). NEDD4 overexpression decreased PMEPA1 expression, whereas co-overexpression of PMEPA1 restored its expression (Figure 7B). Western blot analysis showed a similar pattern at the protein level (Figure 7C).

**FIGURE 7:**
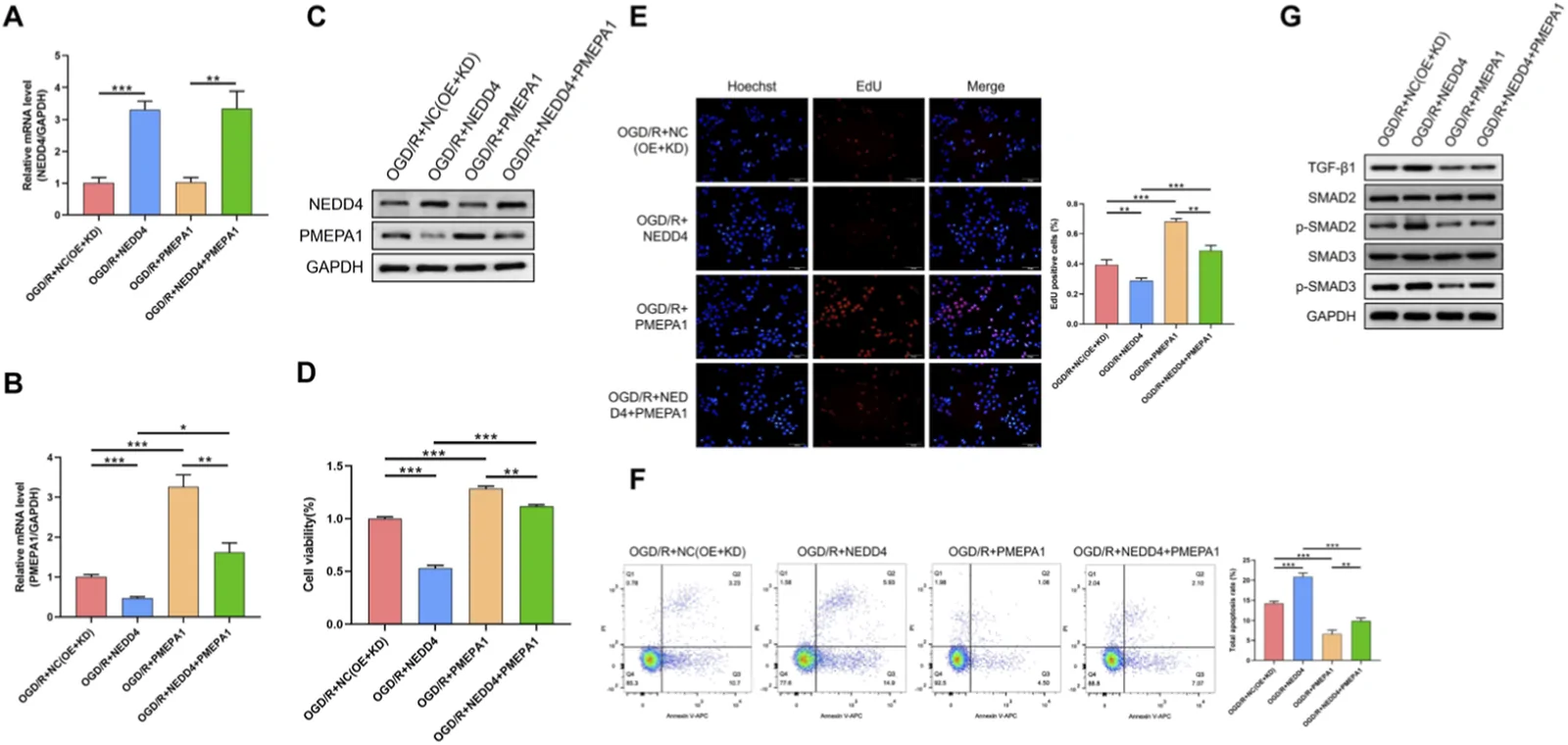
PMEPA1 overexpression attenuates the effects of NEDD4 on cardiomyocyte function and TGF-β/SMAD signaling **(A,B)** qRT-PCR analysis of *NEDD4* **(A)** and *PMEPA1* **(B)** mRNA expression levels in the indicated groups; **(C)** Western blot analysis of NEDD4 and PMEPA1 protein expression in each group; **(D)** Cell viability assessed by CCK8 assay; **(E)** Cell proliferation evaluated by EdU staining; **(F)** Apoptosis analyzed by flow cytometry; **(G)** Western blot analysis of TGF-β pathway–related proteins in each group. Data are from three independent biological replicates (n = 3).

NEDD4 overexpression reduced cell viability and EdU-positive cells and increased apoptosis. PMEPA1 overexpression showed the opposite effects. When PMEPA1 was co-overexpressed with NEDD4, cell viability and proliferation increased, while apoptosis decreased compared with NEDD4 overexpression alone (Figures 7D–F).

NEDD4 overexpression also increased TGF-β1, p-SMAD2, and p-SMAD3 levels. These changes were attenuated by PMEPA1 co-overexpression, while total SMAD2 and SMAD3 levels remained largely unchanged (Figure 7G).

### Activation of TGF-β/SMAD signaling attenuates the effects of PMEPA1 overexpression under OGD/R

PMEPA1 overexpression significantly increased PMEPA1 mRNA and protein levels, and rTGF-β1 treatment (10 ng/mL) did not significantly alter PMEPA1 expression (Figures 8A,B). Under OGD/R conditions, PMEPA1 overexpression increased cell viability and the proportion of EdU-positive cells, while reducing apoptosis. These effects were weakened after rTGF-β1 treatment, with decreased cell viability and proliferation and increased apoptosis compared with the PMEPA1-overexpression group (Figures 8C–E).

**FIGURE 8:**
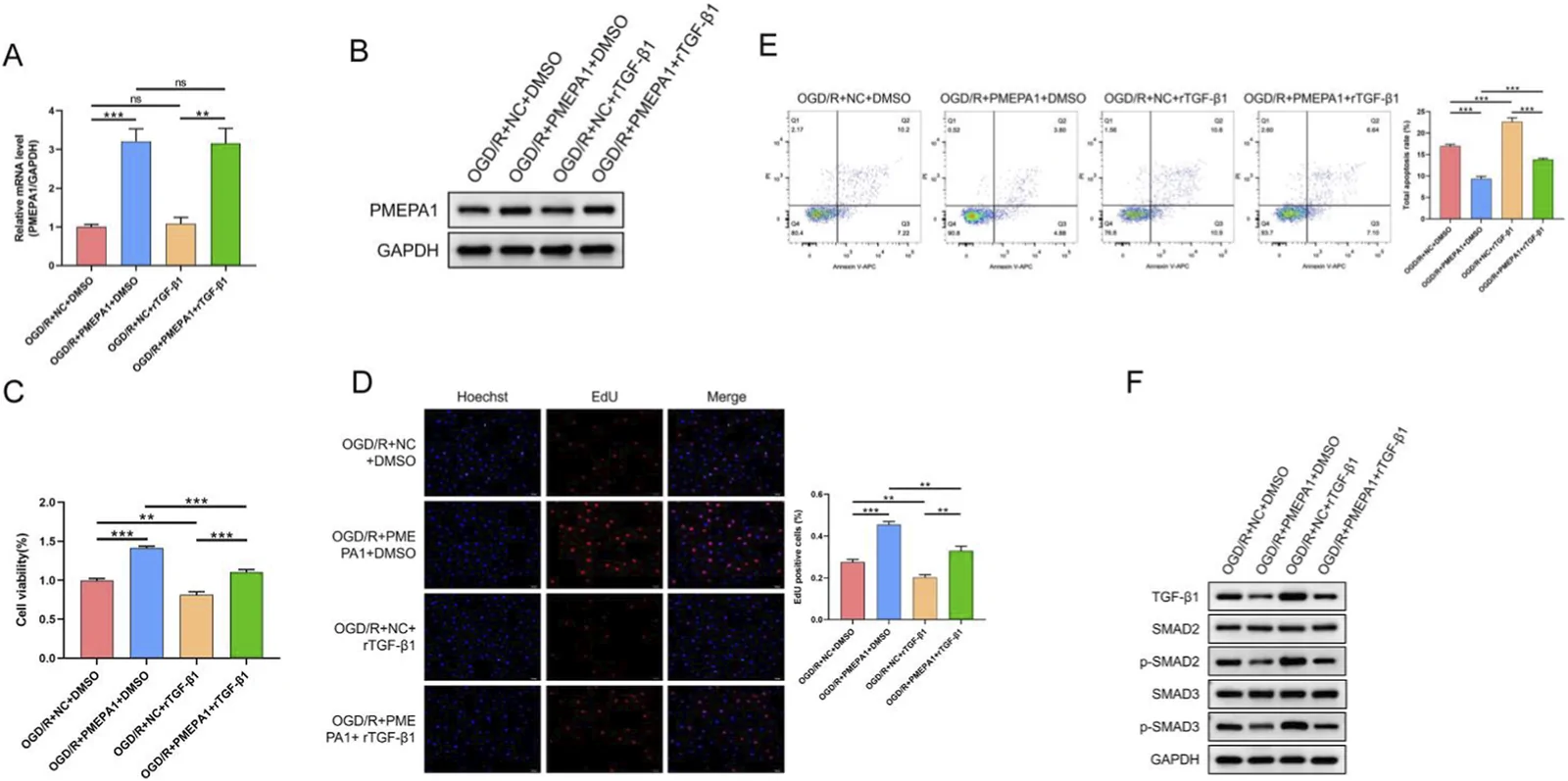
rTGF-β1 attenuates the protective effects of PMEPA1 overexpression under OGD/R conditions. **(A)** qRT-PCR analysis of *PMEPA1* mRNA expression in the indicated groups; **(B)** Western blot analysis of PMEPA1 protein expression; **(C)** Cell viability assessed by CCK-8 assay; **(D)** Cell proliferation evaluated by EdU staining; **(E)** Apoptosis analyzed by flow cytometry; **(F)** Western blot analysis of TGF-β/SMAD pathway-related proteins. Data are from three independent biological replicates (n = 3).

PMEPA1 overexpression also reduced p-SMAD2 and p-SMAD3 levels. After rTGF-β1 treatment, p-SMAD2 and p-SMAD3 were increased again, while total SMAD2 and SMAD3 showed little change (Figure 8F).

### Identification of the key domain mediating the interaction between NEDD4 and PMEPA1

To identify the key domain responsible for the interaction between NEDD4 and PMEPA1, we predicted the putative binding regions of PMEPA1 based on information from the UniProtKB database and relevant literature (Figure 9A). A series of HA-tagged PMEPA1 truncation mutants, each containing different segments of the protein, and a full-length Flag-tagged NEDD4 construct were generated and co-transfected into 293T cells.

**FIGURE 9:**
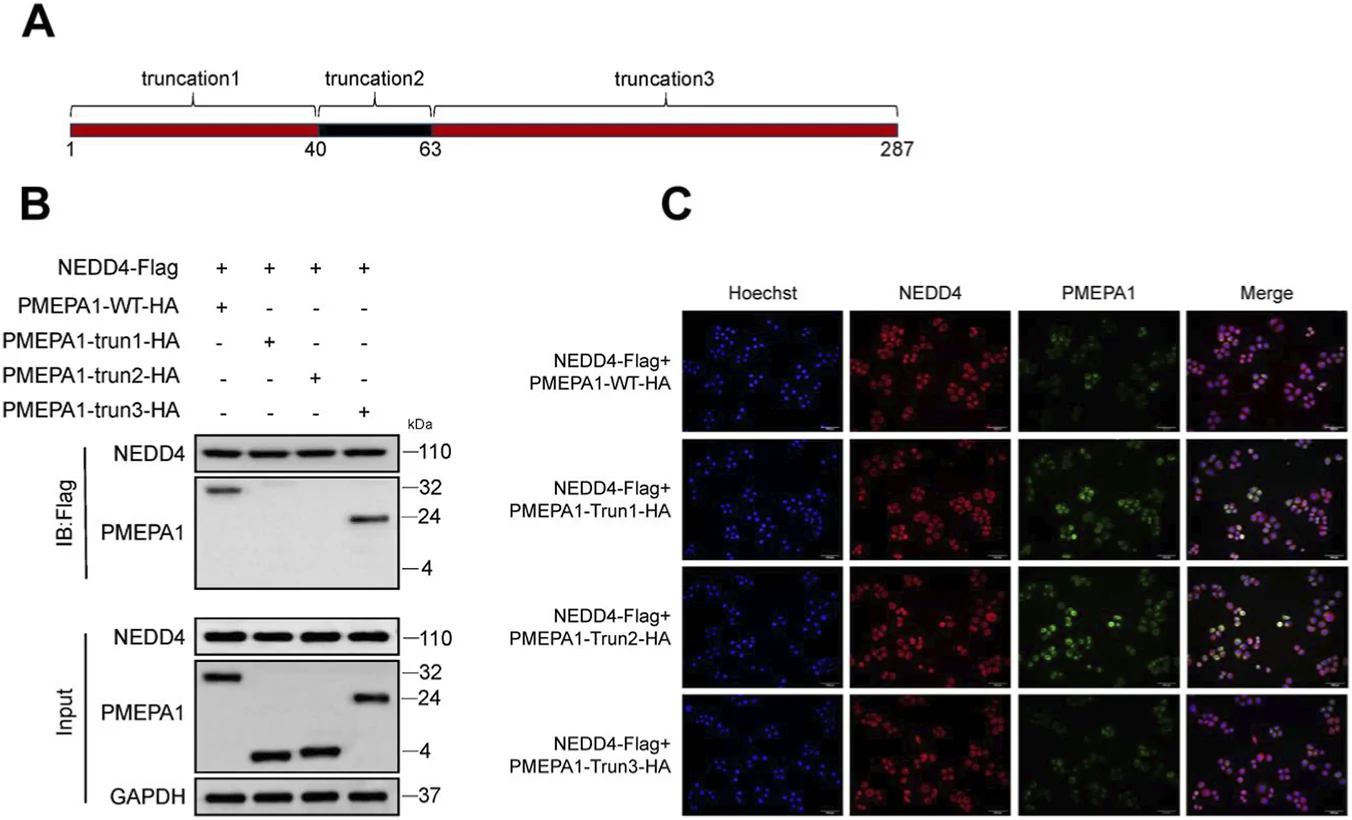
Mapping of the interaction domain between NEDD4 and PMEPA1. **(A)** Schematic representation of PMEPA1 truncated fragments; **(B)** Co-IP analysis of the interaction between NEDD4 and different PMEPA1 fragments; **(C)** Immunofluorescence analysis of protein expression and subcellular colocalization. Data are from three independent biological replicates (n = 3).

Co-ip assays demonstrated that only the PMEPA1 truncation containing the truncation 3 domain was able to interact with NEDD4 (Figure 9B). Consistently, immunofluorescence analysis confirmed clear intracellular colocalization between the truncation 3 domain of PMEPA1 and NEDD4 (Figure 9C).

## Discussion

PMEPA1 is a key regulatory factor in the TGF-β signaling pathway and is involved in a variety of biological processes. Previous research has primarily focused on its roles in cancer and bone metabolism, where it is generally recognized to influence cell proliferation, migration, and differentiation by modulating TGF-β signaling (Fournier et al., 2015; Morikawa et al., 2016; Duncan, 2011). However, the function of PMEPA1 in cardiovascular diseases, particularly in the context of MIRI, has yet to be systematically investigated.

In this study, we performed an integrated analysis of two RNA-seq datasets and applied multiple machine learning algorithms, including LASSO, SVM, and random forest, to screen candidate genes. Both PMEPA1 and PPM1B were identified as hub genes. We focused on PMEPA1 because it is a known negative regulator of TGF-β signaling, which is closely involved in myocardial fibrosis, cardiac remodeling, and cardiac dysfunction. In addition, PMEPA1 expression was significantly downregulated in MIRI, further suggesting its potential role in myocardial injury. However, the role of PMEPA1 in MIRI has not been systematically investigated. Therefore, PMEPA1 was selected for further functional validation.

In both animal and cell models, we found that PMEPA1 expression was significantly downregulated in MIRI and in H9c2 cells exposed to I/R injury. Functional assays demonstrated that overexpression of PMEPA1 promoted cardiomyocyte proliferation, inhibited apoptosis, and reduced the expression of TGF-β1, p-SMAD2, and p-SMAD3, suggesting a potential protective effect in cardiac tissue. Notably, this tissue-protective effect contrasts with observations in certain cancer models, where elevated PMEPA1 expression has been associated with disease progression (Zhang et al., 2019; Pique-Gili et al., 2024; Yang et al., 2022). Such differences likely reflect the unique features of the cardiac microenvironment, cellular composition, and tissue-specific regulation of the TGF-β pathway.

Previous studies have shown that the TGF-β pathway exerts distinct effects at different stages of cardiac injury: it primarily mediates apoptosis and inflammation during the early phase (Schneiders et al., 2005; Heger et al., 2011), while at later stages it promotes post-infarction fibrosis and ventricular remodeling (Liang et al., 2018; Liang et al., 2012; Tan et al., 2010). Our study focused on the early injury phase and demonstrated that PMEPA1 overexpression effectively reduced cardiomyocyte apoptosis, in line with studies implicating the TGF-β/SMAD pathway in early myocardial injury. Collectively, these findings suggest that PMEPA1 may confer cardioprotection against MIRI by negatively regulating TGF-β/SMAD signaling at an early stage. Nevertheless, further investigation is required to delineate the precise molecular mechanisms and context-dependent roles of PMEPA1 across different diseases and time points.

To gain insight into the upstream regulation of PMEPA1 in myocardial injury, we examined potential interacting partners. Bioinformatic predictions using the STRING database identified NEDD4, an E3 ubiquitin ligase, as a strong candidate. This was supported by prior studies showing that NEDD4 can modulate the stability and function of various substrate proteins through ubiquitination in disease models involving the nervous system and cancer (Wang et al., 2020; Tian et al., 2023; Sugeno et al., 2014; Tofaris et al., 2011).

In recent years, the role of NEDD4 in the cardiovascular system has received increasing attention. Studies have shown that NEDD4 can attenuate myocardial injury by inhibiting NLRP3 inflammasome activation and reducing macrophage pyroptosis (Sun et al., 2023). In this study, direct protein–protein interaction between NEDD4 and PMEPA1 was confirmed by co-ip and immunofluorescence colocalization assays. Functional experiments revealed that both NEDD4 overexpression and PMEPA1 knockdown aggravated cardiomyocyte injury and enhanced activation of the TGF-β/SMAD signaling pathway. Notably, the effect of NEDD4 overexpression on cell injury and pathway activation was diminished when PMEPA1 was downregulated, suggesting that the regulatory function of NEDD4 may depend on PMEPA1 expression levels. Taken together, these findings suggest that NEDD4 may act as an upstream regulator by modulating PMEPA1 expression and thereby participate in TGF-β/SMAD pathway–mediated myocardial injury.

It should be noted that several limitations remain in this study. Although an interaction between NEDD4 and PMEPA1 was identified, direct evidence of NEDD4-mediated ubiquitination of PMEPA1 is still lacking. In addition, the findings were not validated in clinical samples, and the *in vitro* experiments were mainly performed in H9c2 cells. Although the animal model provided *in vivo* evidence for the expression changes of PMEPA1, the cellular findings were not further validated in primary cardiomyocytes. Future studies using primary cardiomyocytes are therefore needed to confirm the physiological relevance of these findings. Moreover, although changes in PMEPA1 expression were confirmed in the MIRI rat model, its protective role has not yet been directly verified *in vivo* by PMEPA1 overexpression or knockdown, infarct size assessment, or cardiac function evaluation. Further studies using direct ubiquitination assays, clinical samples, primary cardiac cells, and *in vivo* functional validation are needed to confirm and extend these findings.

In summary, this study clarified the potential roles of PMEPA1 and its upstream regulator NEDD4 in MIRI, expanding our understanding of the TGF-β signaling network. These findings provide new theoretical insights into the molecular mechanisms underlying cardiovascular injury and suggest possible targets for intervention. Given the critical role of the TGF-β pathway in cardiac remodeling and various chronic cardiovascular diseases, the NEDD4–PMEPA1 regulatory axis may represent a promising avenue for future precision therapies in cardiovascular medicine.

## Data Availability

Publicly available datasets were analyzed in this study. This data can be found here: Gene Expression Omnibus, accession numbers GSE127853 and GSE194388.
