# Supplementary material for: PMEPA1 attenuates myocardial ischemia-reperfusion injury via interaction with NEDD4 and involvement of the TGF-β/SMAD pathway
Source: Front Pharmacol. 2026 Oct 1;17:1881951. doi: 10.3389/fphar.2026.1881951 (PMC13668540; doi:10.3389/fphar.2026.1881951)
Supplement: Supplementary file 1 [file Supplementaryfile1.docx]

**PMEPA1 attenuates myocardial ischemia-reperfusion injury via interaction with NEDD4 and involvement of the TGF-β/SMAD pathway**

Weilong Zhong ^1†^, Yanan Li ^2†^ , Baoze Qu ^1^*

^1^The First Affiliated Hospital of Jinzhou Medical University

^2^Jinzhou Maternal and Child Hospital

**†**These authors contributed equally to this work.

*Corresponding author: Qu Baoze, The First Affiliated Hospital of Jinzhou Medical University, Renmin Street, Jinzhou, Liaoning 121001, China. E-Mail:qubz@jzmu.edu.cn

**Supplementary Table 1 The primers of the detected genes**

| **gene** |  | **Primer（5’-3’）** |
| --- | --- | --- |
| PMEPA1 | Forward | GTCTGCACGGTCCTTCATCA |
|  | Reverse | CGTTGCCTGACACTGTGCTC |
| GAPDH | Forward | ACCACAGTCCATGCCATCAC |
|  | Reverse | TCCACCACCCTGTTGCTGTA |

**Supplementary Table 2 Information of utilized antibodies**

| **Primary antibodies** | | | |
| --- | --- | --- | --- |
| **Antibody** | **Manufacturer** | **Cat No.** | **Dilution** |
| PMEPA1 | Proteintech | 16521-1-AP | **WB**(1:1000), **IHC(**1:500**)** |
| TGF-β1 | Cell Signaling Technology | #3711 | **WB**(1:1000) |
| SMAD2 | Cell Signaling Technology | #5339 | **WB**(1:1000) |
| p-SMAD2 | Cell Signaling Technology | #3108 | **WB**(1:1000) |
| SMAD3 | Cell Signaling Technology | #9523 | **WB**(1:1000) |
| p-SMAD3 | Cell Signaling Technology | #9520 | **WB**(1:1000) |
| GAPDH | Proteintech | 10494-1-AP | **WB**(1:5000) |

| **Secondary antibodies** | | | |
| --- | --- | --- | --- |
| **Antibody** | **Manufacturer** | **Cat No.** | **Dilution** |
| goat anti-rabbit IgG | Beyotime | A0208 | 1:1000 |

**Supplemental Table 3. The target sequences of shRNA**

| **Target gene** | **TRC Clone ID** | **Target region** | **Target sequence** |
| --- | --- | --- | --- |
| PMEPA1 | TRCN0000272493 | CDS | TGCAACTGCAAACGCTCTTTG |

**Supplemental Table 4. Differentially expressed genes (DEGs) identified in GSE127853 and GSE194388.**

| id | logFC | AveExpr | t | P.Value | adj.P.Val |
| --- | --- | --- | --- | --- | --- |
| TPM1 | 1.89066102038408 | 4.90920021912259 | 7.17619159316496 | 1.05117020909134e-07 | 0.000674712623106756 |
| PLA2G12A | 1.54202376991392 | 3.48739681634522 | 7.10431567593981 | 1.25879220728872e-07 | 0.000674712623106756 |
| IFNG | -2.368467765 | 1.9948386891581 | -6.310176343 | 9.59241479126581e-07 | 0.00342768955207898 |
| PDE5A | 1.73829002459658 | 4.47317178334079 | 6.05742405693572 | 1.8559703684745e-06 | 0.00367368256247771 |
| ENDOD1 | 1.77822480654157 | 3.72759974024038 | 6.05365580741183 | 1.87440542706213e-06 | 0.00367368256247771 |
| NPTN | 1.5906154146402 | 4.93269488279556 | 6.01839280363184 | 2.05616561332708e-06 | 0.00367368256247771 |
| TMEM55A | 1.48036830720547 | 3.83227423888461 | 5.90118963954743 | 2.79882766176712e-06 | 0.0042862046477348 |
| TWSG1 | 1.65413506299544 | 1.86425675498418 | 5.62104576164462 | 5.87365364585427e-06 | 0.00771668118456769 |
| FOXO1 | -1.23244268 | 3.72544675687978 | -5.570533502 | 6.71738154253859e-06 | 0.00771668118456769 |
| NR1D1 | -1.591767828 | 1.97360616423705 | -5.514000707 | 7.807601112743e-06 | 0.00771668118456769 |
| VCL | 1.53971029351448 | 6.03010413938849 | 5.47975039381315 | 8.55319248515546e-06 | 0.00771668118456769 |
| ADAM19 | -1.079732024 | 2.55678850807011 | -5.476043433 | 8.63807595287428e-06 | 0.00771668118456769 |
| AIG1 | 1.45212040637641 | 3.33431841752427 | 5.3904411672886 | 1.08527528220529e-05 | 0.00838525099312633 |
| NT5C3A | 1.28244600751151 | 6.12058130541204 | 5.36901140099732 | 1.14915527748854e-05 | 0.00838525099312633 |
| MMD | 1.61863057730524 | 4.98606748198665 | 5.35053882368812 | 1.2072500017133e-05 | 0.00838525099312633 |
| RAB27B | 1.5382761716782 | 4.0538704418118 | 5.31675888960634 | 1.32123790535714e-05 | 0.00838525099312633 |
| GRASP | -1.497251178 | 3.06031542895629 | -5.314355061 | 1.32975062390996e-05 | 0.00838525099312633 |
| ITGB1 | 1.47653305377825 | 6.49691425828801 | 5.2750914775768 | 1.47687047991033e-05 | 0.00879558419146599 |
| ANO6 | 1.46583281559343 | 5.10335237398641 | 5.22041994696429 | 1.70938797241602e-05 | 0.0092398169569432 |
| PDE8A | -1.123533185 | 3.15918344135098 | -5.171813875 | 1.94684553062264e-05 | 0.0092398169569432 |
| TGIF2 | -1.350540125 | 2.19117834774527 | -5.169789165 | 1.95742597602634e-05 | 0.0092398169569432 |
| SNHG7 | -1.306024783 | 2.68026790554861 | -5.153372528 | 2.04537369802816e-05 | 0.0092398169569432 |
| ANKRD28 | 1.2765677215877 | 4.44422180114797 | 5.14512462603181 | 2.09104616533125e-05 | 0.0092398169569432 |
| PTGER4 | -1.272545486 | 2.860536205 | -5.129879234 | 2.17817945788914e-05 | 0.0092398169569432 |
| ACTR3B | 1.35523378774217 | 1.85653336746517 | 5.11005310801837 | 2.29696815927107e-05 | 0.0092398169569432 |
| FASLG | -1.430898942 | 2.57639125008967 | -5.105424671 | 2.32562289239903e-05 | 0.0092398169569432 |
| SLC15A3 | -1.261677524 | 2.59289633173819 | -5.105172764 | 2.32719270370771e-05 | 0.0092398169569432 |
| KIF2A | 1.39901856270235 | 6.62796448723002 | 5.06721080685028 | 2.57633487033632e-05 | 0.00937447659581458 |
| YWHAH | 1.16731902382914 | 6.33165949161573 | 5.06559064936399 | 2.58754389713005e-05 | 0.00937447659581458 |
| RSU1 | 1.57474432512909 | 6.35262019132354 | 5.06044714485207 | 2.62345427121677e-05 | 0.00937447659581458 |
| ATP13A3 | -1.044657893 | 2.56308266972137 | -5.002990347 | 3.06022854077024e-05 | 0.0105824677280829 |
| PPM1B | -1.098454917 | 4.73144841935016 | -4.991036721 | 3.15988317190483e-05 | 0.0105856086258812 |
| NR1D2 | -1.080207426 | 2.97555177700249 | -4.947166875 | 3.55430016181833e-05 | 0.0115460902226341 |
| ITM2B | 1.25659940114494 | 6.1387160211889 | 4.91949286507523 | 3.82809695563664e-05 | 0.0120106326293933 |
| DAAM1 | 1.22414591649798 | 3.52585762888307 | 4.89997278443913 | 4.0338275697619e-05 | 0.0120106326293933 |
| TSC22D1 | 1.44978440854717 | 5.05585029173458 | 4.89947648142248 | 4.03920012540035e-05 | 0.0120106326293933 |
| TUBB1 | 1.7287322363603 | 6.54177707959517 | 4.88979382090527 | 4.14546088887643e-05 | 0.0120106326293933 |
| ARRDC2 | -1.191398497 | 4.31047006945878 | -4.859229308 | 4.49958568872145e-05 | 0.0126935680481826 |
| ZNF699 | -1.056676439 | 2.14822542239443 | -4.843897234 | 4.68846542019613e-05 | 0.0128232415120393 |
| SESN2 | -1.431503934 | 1.52208131048203 | -4.835110396 | 4.80026582953447e-05 | 0.0128232415120393 |
| TSPAN33 | 1.50085839102935 | 3.67404055070969 | 4.82710735997888 | 4.90441139919414e-05 | 0.0128232415120393 |
| HEG1 | -1.12424346 | 2.37202100062312 | -4.802000434 | 5.24602060200571e-05 | 0.0133898430603574 |
| NEK6 | -1.095175566 | 2.92702471350696 | -4.767442545 | 5.75544599051467e-05 | 0.0140223593223448 |
| SNAPC3 | -1.105141223 | 2.33194362936534 | -4.747137734 | 6.07751279038757e-05 | 0.0141365608295726 |
| ZC3H12A | -1.671113275 | 2.56366086818486 | -4.725118123 | 6.4471503766831e-05 | 0.0142638892097064 |
| GAB2 | -1.084947652 | 3.46109442936373 | -4.720934599 | 6.51987473204862e-05 | 0.0142638892097064 |
| HELZ2 | -1.005468668 | 1.29497089086343 | -4.689512886 | 7.09291131389621e-05 | 0.0149494979013702 |
| SNAP23 | 1.21364239260882 | 5.97936459886612 | 4.68850151143864 | 7.11216784486827e-05 | 0.0149494979013702 |
| PRUNE1 | 1.28852742453184 | 4.16512566894934 | 4.674282195 | 7.38849008584027e-05 | 0.0152316564846553 |
| CD226 | 1.34473095905728 | 4.31889938659588 | 4.65572567540801 | 7.76526457294962e-05 | 0.0157063464569849 |
| F2R | 1.1704792369488 | 3.61699334903558 | 4.62098987310916 | 8.52279312299046e-05 | 0.0163775122563572 |
| RAPGEF1 | -1.334617938 | 4.83373111040054 | -4.619563964 | 8.55541685033584e-05 | 0.0163775122563572 |
| KDM6B | -1.481620329 | 3.32927762763428 | -4.611983679 | 8.73094890118936e-05 | 0.016420310915921 |
| OST4 | 1.3925041454638 | 10.0212549619524 | 4.60263921063414 | 8.95227953982431e-05 | 0.0165462821839511 |
| PUS10 | -1.154767206 | 1.30140258146381 | -4.591025621 | 9.23516267810245e-05 | 0.0166061762599402 |
| APP | 1.48977121616779 | 4.67701395009549 | 4.58471990960236 | 9.39246899840567e-05 | 0.0166061762599402 |
| CDK5 | -1.71002467 | 1.50072739616491 | -4.579751606 | 9.51828908911294e-05 | 0.0166061762599402 |
| SERPINB9 | -1.207322767 | 4.93743671551817 | -4.576392049 | 9.60431835929378e-05 | 0.0166061762599402 |
| CNST | 1.29823095524484 | 4.49152854659424 | 4.53816888082421 | 0.000106393540117792 | 0.0172808901524655 |
| FBXL13 | 1.27094786392113 | 1.73009712159023 | 4.52731009341557 | 0.000109531471335586 | 0.0175250354136938 |
| CLU | 1.88305808608622 | 6.07329659562165 | 4.51930127594566 | 0.0001119046868876 | 0.0176414447563981 |
| ZNF304 | -1.176210314 | 1.44676451895758 | -4.505353365 | 0.000116160582607453 | 0.0180469774717666 |
| VIM-AS1 | 1.63706934669056 | 4.57933807481493 | 4.49127825060909 | 0.000120618502830821 | 0.0184718621478057 |
| EVA1C | 1.40613510476888 | 2.12729114761992 | 4.46995708748533 | 0.000127697529935734 | 0.0192805284635362 |
| CTSA | 1.35824230955943 | 5.89125582321463 | 4.45567528021036 | 0.000132668849318259 | 0.0194384600209837 |
| ZBTB21 | -1.041255335 | 1.59711291992036 | -4.449719706 | 0.000134798315362276 | 0.0194384600209837 |
| PMEPA1 | -1.05093492 | 0.937928751618879 | -4.446409146 | 0.000135996688579643 | 0.0194384600209837 |
| ISG20L2 | -1.106445425 | 3.64780757054092 | -4.402677421 | 0.000152856653023676 | 0.0210079912873565 |
| PHLDB2 | -1.190721036 | 2.46819101988459 | -4.360427611 | 0.00017111225041146 | 0.0227095913670974 |
| ATG7 | -1.135285208 | 2.51472970384068 | -4.35649133 | 0.000172919454696278 | 0.0227095913670974 |
| POLB | -1.18226662 | 3.89344127743622 | -4.354779179 | 0.000173711426502051 | 0.0227095913670974 |
| MFSD1 | 1.12658771928817 | 5.18057794705271 | 4.338533485 | 0.000181407378605017 | 0.0231350528699966 |
| ANPEP | -1.34807273 | 2.27468587500337 | -4.334356332 | 0.000183440251301279 | 0.0231350528699966 |
| LEPROT | 1.19504759867894 | 4.08567873660896 | 4.32600237497188 | 0.000187573840622733 | 0.0233055785087571 |
| NOL9 | -1.041527526 | 2.28468803906351 | -4.320254467 | 0.000190471446851631 | 0.0233055785087571 |
| B2M | 1.39346798386549 | 11.8080179051323 | 4.31473997376359 | 0.000193293085100639 | 0.0233055785087571 |
| IER3 | -1.81365428 | 2.25492136039517 | -4.302258075 | 0.000199833748666467 | 0.0237928353008511 |
| PF4 | 1.93397949650986 | 9.66126336361694 | 4.29010687309463 | 0.000206411724158503 | 0.0237928353008511 |
| RAB11A | 1.0497029105674 | 5.97150986817633 | 4.2829644580773 | 0.000210377884319631 | 0.0238654718442977 |
| VAMP7 | 1.09636760267737 | 3.17408930820273 | 4.28097787468536 | 0.000211494386679877 | 0.0238654718442977 |
| HERC5 | -1.120690281 | 2.66686339889436 | -4.26445502 | 0.000221010929431197 | 0.024070829923268 |
| CTNS | 1.1272897205112 | 1.16490348541541 | 4.25849322486582 | 0.000224547823198372 | 0.024070829923268 |
| CXCR2P1 | 1.40646368454215 | 5.29180394956355 | 4.25584905289416 | 0.000226134393480076 | 0.024070829923268 |
| NFKBID | -1.554734584 | 2.4872404603228 | -4.254767185 | 0.000226786737150193 | 0.024070829923268 |
| RGS10 | 1.09869701740887 | 7.94800807177962 | 4.24772247030898 | 0.000231080361846705 | 0.024212771259732 |
| PLEKHM2 | -1.212176419 | 2.96755077308257 | -4.232849154 | 0.0002404114673123 | 0.0245448659960748 |
| CD6 | -1.245340878 | 3.2331660235248 | -4.219387829 | 0.000249177724104069 | 0.0251998603999587 |
| AVPI1 | -1.546134427 | 1.23372728102613 | -4.202359246 | 0.000260721418319956 | 0.0257665442704018 |
| BCAP31 | 1.18999313073717 | 5.39513630432778 | 4.20231437818528 | 0.000260752522668342 | 0.0257665442704018 |
| PDLIM1 | 1.36387785294196 | 6.3109442693949 | 4.19723182299789 | 0.000264299813268771 | 0.0257665442704018 |
| RDH11 | 1.44676503469004 | 4.0451986165354 | 4.16747484619647 | 0.000286046575878869 | 0.0269404532949269 |
| CD83 | -1.613152752 | 2.94579991319903 | -4.162522225 | 0.000289833876015736 | 0.0269404532949269 |
| ANKRD37 | -1.679186169 | 1.56909442641818 | -4.160126636 | 0.000291683540233677 | 0.0269404532949269 |
| ELOVL7 | 1.60299464551715 | 3.65950225076018 | 4.15756705191095 | 0.000293672719600942 | 0.0269404532949269 |
| ZNF227 | -1.129840507 | 2.09690036792219 | -4.15710538 | 0.000294032932416646 | 0.0269404532949269 |
| RGS1 | -1.622004958 | 2.32293044521186 | -4.129062399 | 0.000316751517426118 | 0.0283026567697401 |
| DTX2 | -1.407992508 | 2.68040750237297 | -4.111894367 | 0.000331506678426136 | 0.0284300127418254 |
| BHLHE40 | -1.434040568 | 4.38050811104015 | -4.100746395 | 0.000341449165818817 | 0.0290502782347438 |
| BCL3 | -1.523501148 | 3.71841091777135 | -4.08714283 | 0.000353981126848854 | 0.029879351809604 |
| ZNF133 | -1.119729288 | 1.45754790058995 | -4.07580833 | 0.000364768547708039 | 0.0305493658705483 |
| FEZ1 | -1.473265087 | 2.76021271372487 | -4.052478369 | 0.000388003371806048 | 0.0309907278344165 |
| PLCL2 | -1.173673772 | 4.46485162539317 | -4.052257972 | 0.000388229685989074 | 0.0309907278344165 |
| NBEAL1 | -1.25620136 | 3.89521582505636 | -4.049437893 | 0.000391136983512589 | 0.0309907278344165 |
| PRCP | -1.047810778 | 2.4505450559376 | -4.044853467 | 0.000395909070898319 | 0.0309907278344165 |
| IGFBP6 | -1.616093355 | 1.67318127297444 | -4.043851526 | 0.000396959644684642 | 0.0309907278344165 |
| NRARP | -1.647854957 | 1.12057981830018 | -4.040197308 | 0.000400814573409859 | 0.0309907278344165 |
| PIGT | -1.258832733 | 2.88693028833046 | -4.036521358 | 0.000404729654553947 | 0.0309907278344165 |
| CENPU | 1.25998059287827 | 1.90616433008983 | 4.01602035495841 | 0.000427265512529471 | 0.0320299740861254 |
| SDPR | 1.52489888137536 | 6.69703875900356 | 4.00403659099604 | 0.000441006643282852 | 0.0326566503253273 |
| SNX20 | -1.003425705 | 4.36204773396114 | -4.003426479 | 0.000441717751601908 | 0.0326566503253273 |
| TNFRSF1A | -1.226808144 | 3.32376100411748 | -3.982716529 | 0.000466537288638279 | 0.0337924306365024 |
| SKIL | -1.163609117 | 3.24197732122047 | -3.978954811 | 0.000471190554697287 | 0.0338024676731747 |
| KLF12 | -1.047716715 | 3.84658907845155 | -3.977516108 | 0.000472982290203005 | 0.0338024676731747 |
| CDK9 | -1.103437643 | 3.50340195138527 | -3.961684351 | 0.000493147224900458 | 0.0343281704606033 |
| TTC33 | 1.27029409571293 | 2.72519175532662 | 3.9578725086368 | 0.000498127359723827 | 0.0344511309434802 |
| ZNF529-AS1 | 1.17274791991812 | 1.34229941829303 | 3.94339709098849 | 0.000517494086238227 | 0.0355611320799602 |
| S1PR2 | -1.006628943 | 1.20754162751214 | -3.940436421 | 0.000521545458539387 | 0.0356112567868932 |
| JUND | -1.004076947 | 5.81738716461768 | -3.925158395 | 0.000542952767043012 | 0.0366066268094408 |
| TRERF1 | -1.124947239 | 2.81297365340118 | -3.920019595 | 0.000550345509307383 | 0.0366915938412131 |
| NLK | 1.22203703910126 | 3.92822373018605 | 3.91050510514104 | 0.000564295258591584 | 0.0366915938412131 |
| FOXN3 | -1.030348614 | 4.72187187413622 | -3.908295741 | 0.000567583922746255 | 0.0366915938412131 |
| PLK2 | -1.239173975 | 3.21303028811763 | -3.90822172 | 0.000567694428611003 | 0.0366915938412131 |
| LSM11 | -1.106802951 | 0.913904420677291 | -3.905647903 | 0.000571550076211217 | 0.0366915938412131 |
| SMG6 | -1.070088298 | 2.69615239239318 | -3.90109061 | 0.00057844023872808 | 0.0366915938412131 |
| DDIT4 | -1.0249973 | 4.84776382579716 | -3.895661884 | 0.000586754480500313 | 0.0368014305940933 |
| LINC01011 | 1.87317142089613 | 2.6014717699315 | 3.8925233521563 | 0.000591614664763952 | 0.0368014305940933 |
| SOCS1 | -1.338875593 | 2.49725722477249 | -3.891053949 | 0.000593903684027812 | 0.0368014305940933 |
| SLC31A1 | -1.103226353 | 2.2032590636006 | -3.888380626 | 0.000598090487352027 | 0.0368478737035272 |
| TGFBR1 | -1.059722665 | 2.10810094538568 | -3.885410438 | 0.000602776224635972 | 0.036924349303415 |
| IFIH1 | -1.229162861 | 3.24517996627197 | -3.872176777 | 0.000624095403561125 | 0.0377983204868659 |
| TRAF4 | -1.284228476 | 1.68771581706113 | -3.865203548 | 0.000635625199851751 | 0.0382237771706851 |
| EFHC2 | 1.47317577778598 | 2.34648057273916 | 3.86363252779814 | 0.00063825150312991 | 0.0382237771706851 |
| SMAD7 | -1.08219875 | 2.42995678197684 | -3.861478234 | 0.000641870199077074 | 0.0382269363005902 |
| TNF | -1.791169179 | 2.86598699307463 | -3.833996836 | 0.000689837009941169 | 0.0408120851881213 |
| SERPINF1 | -1.30149699 | 2.23283537467667 | -3.827717075 | 0.000701281944448197 | 0.0408120851881213 |
| IFIT2 | -1.110628867 | 3.52733515237524 | -3.82204348 | 0.000711782113506591 | 0.0410231411655411 |
| BEX3 | 1.80911771808284 | 6.4310269630427 | 3.8140312232701 | 0.000726873459062098 | 0.0416478244366627 |
| PDE4A | -1.315492597 | 2.57006543978115 | -3.805721693 | 0.000742856009342916 | 0.0416478244366627 |
| L2HGDH | -1.060578297 | 1.5324345700604 | -3.804085553 | 0.000746043303350058 | 0.0416478244366627 |
| YES1 | -1.21888155 | 3.51893702181039 | -3.801368683 | 0.000751365548935202 | 0.0416478244366627 |
| PCYT1B | 1.07029946434062 | 2.22571949465052 | 3.79185008804611 | 0.000770307279195256 | 0.0419172285937723 |
| NFKB1 | -1.074340627 | 4.54561095869574 | -3.77860604 | 0.000797442886984598 | 0.0425302873058452 |
| DENND2A | -1.186877587 | 1.1942699773285 | -3.774967654 | 0.000805060040903768 | 0.0425962167199813 |
| BMF | -1.066234172 | 2.00964745454748 | -3.757759443 | 0.00084206248560674 | 0.0440292729641783 |
| GRINA | -1.234438349 | 4.13309906528635 | -3.738639116 | 0.000885130142644227 | 0.0449696451618299 |
| ZNF347 | -1.007107047 | 2.04382455056981 | -3.722182241 | 0.000923918319210623 | 0.0453656232865277 |
| AKTIP | -1.136508688 | 3.31718585802987 | -3.716258418 | 0.000938283435647407 | 0.0457199928642736 |
| KLF16 | -1.056230917 | 2.21255663826172 | -3.69849313 | 0.000982685569210388 | 0.0460694239795172 |
| UGCG | -1.116691617 | 2.38016887746099 | -3.691901197 | 0.000999678238109043 | 0.0460694239795172 |
| JAM3 | 1.62614122682256 | 4.14399279493737 | 3.67920349200852 | 0.00103322318124384 | 0.0462217629320405 |
| NRM | -1.074749303 | 1.99812330684181 | -3.665724956 | 0.00107003358107965 | 0.0472047736179994 |
| ZNF585B | -1.084244204 | 1.8883885978979 | -3.660007389 | 0.00108603244032643 | 0.0473575091407853 |
| SEMA4D | 1.11406202414679 | 5.32618038881848 | 3.65371427673268 | 0.00110391175016282 | 0.0473575091407853 |
| PGRMC1 | 1.6301184075306 | 6.00417493124402 | 3.65287672532296 | 0.00110631286547281 | 0.0473575091407853 |
| ERCC1 | -1.054774861 | 3.24416428469288 | -3.645510306 | 0.00112765182179479 | 0.0477803459669573 |
| RAMP2-AS1 | -1.063467296 | 1.64591353533653 | -3.63550201 | 0.00115728825957144 | 0.0488430320575032 |
| BTG2 | -1.003563296 | 5.42447430148403 | -3.625277522 | 0.0011883490345567 | 0.0496733982569251 |
| ZNF37A | -1.009531427 | 2.24858299366249 | -3.618552777 | 0.00120921958675058 | 0.0500495520075915 |
| CCL5 | 1.04328815871846 | 10.1743640683113 | 3.61251097013727 | 0.00122827505391013 | 0.0500989501849895 |
| RELB | -1.25840865 | 3.467267738 | -3.607856965 | 0.00124315274530764 | 0.0500989501849895 |
| RAVER1 | -1.005635582 | 2.72937838880838 | -3.607630691 | 0.0012438805474195 | 0.0500989501849895 |
| TMSB4X | 1.0127728025204 | 12.379200567135 | 3.60641423690798 | 0.00124780034509256 | 0.0500989501849895 |
| RBX1 | 1.13872898379526 | 5.42120044191141 | 3.58868241885519 | 0.00130632276107837 | 0.0506105050967918 |
| HBA2 | -1.9909453 | 8.6808801641335 | -3.580070252 | 0.00133570275889649 | 0.050611088396365 |
| FOSL2 | -1.033646012 | 3.78422753340866 | -3.571593792 | 0.00136524765035951 | 0.0511729189225662 |
| DUSP2 | -1.266731113 | 3.01443592920977 | -3.56640839 | 0.00138363410077286 | 0.0516813852274742 |
| MAP3K7CL | 1.3410941962578 | 6.46667463126628 | 3.56174549334101 | 0.00140037349387806 | 0.0519446500151309 |
| TBC1D22B | -1.137378782 | 2.50817968034285 | -3.558815736 | 0.00141099171376648 | 0.0521580385226782 |
| PLAGL2 | -1.058022692 | 3.02916976320826 | -3.549559776 | 0.00144505503730361 | 0.0530513356160779 |
| C6orf25 | 1.38491154573133 | 5.30513305196596 | 3.54131090425565 | 0.00147608528315844 | 0.0538218851546206 |
| NEXN | 1.47257625101996 | 4.58032615235645 | 3.5380349336247 | 0.00148858765020508 | 0.0539110121966163 |
| RBMS2 | -1.029948722 | 2.89226441401926 | -3.523894736 | 0.00154374368142586 | 0.0551631075496174 |
| NFYA | -1.057108003 | 3.09889469013089 | -3.509618904 | 0.00160144204794507 | 0.0560470197420125 |
| ANGPT1 | 1.4281062081764 | 2.95595479637632 | 3.50826764060877 | 0.00160701073756255 | 0.0560470197420125 |
| ADA | -1.129931697 | 3.63961073122721 | -3.504226961 | 0.00162377523493119 | 0.0560470197420125 |
| ZNF814 | -1.042025653 | 1.60878944182468 | -3.502358282 | 0.00163158559130305 | 0.0560470197420125 |
| CSF2 | -1.280406843 | 0.988591744321412 | -3.501199421 | 0.00163644749806436 | 0.0560470197420125 |
| GEMIN7 | -1.006149127 | 2.00446729890826 | -3.489476259 | 0.00168642890313947 | 0.0566883562214656 |
| STAT4 | -1.041359162 | 4.52878178828714 | -3.489216358 | 0.00168755360944831 | 0.0566883562214656 |
| ADCK2 | -1.081023244 | 1.33658158000154 | -3.488146799 | 0.0016921897379542 | 0.0566883562214656 |
| GADD45B | -1.146190209 | 4.01748406863267 | -3.484971079 | 0.00170602825394841 | 0.056743591099703 |
| ACRBP | 1.08322639532919 | 5.01550408212273 | 3.4841284996993 | 0.00170971827660486 | 0.056743591099703 |
| IKBIP | -1.001015298 | 2.10069134061919 | -3.472231383 | 0.0017626552534164 | 0.0573360537019127 |
| GPR132 | -1.245516593 | 2.42838594907137 | -3.469661372 | 0.00177429783917125 | 0.0573360537019127 |
| TIPARP | -1.058169633 | 2.41660932511623 | -3.469023955 | 0.00177719697625614 | 0.0573360537019127 |
| RUNX3 | -1.03919579 | 5.0456489881689 | -3.467112304 | 0.00178591924266879 | 0.0573360537019127 |
| MS4A7 | -1.013658574 | 5.12562071424215 | -3.467006508 | 0.00178640316571258 | 0.0573360537019127 |
| POM121C | -1.053463011 | 2.69314757221276 | -3.46363579 | 0.00180188804412007 | 0.0575247332245268 |
| SMYD5 | -1.081822028 | 1.56101972394079 | -3.462412436 | 0.00180754020297403 | 0.0575247332245268 |
| SERPINI1 | 1.21332172264492 | 1.17016438488577 | 3.45250828125163 | 0.00185393706048083 | 0.0584535449657485 |
| RABGGTA | -1.067517161 | 2.42703611484168 | -3.448641792 | 0.00187236164317401 | 0.0584738627937613 |
| OAS3 | -1.027932054 | 2.87455787034987 | -3.447126587 | 0.00187963020805105 | 0.0584738627937613 |
| CLUHP3 | -1.009569061 | 1.37214059797577 | -3.445627735 | 0.0018868472385972 | 0.0584738627937613 |
| NCK1-AS1 | 1.32135934709014 | 2.05820888932513 | 3.44301355346771 | 0.00189949896229382 | 0.0586614426748725 |
| PRKAR1B | 1.05056612004107 | 2.63844332390935 | 3.43940973633694 | 0.00191707507661181 | 0.0586614426748725 |
| PPBP | 1.42016934094944 | 9.9523967373185 | 3.43850945168461 | 0.00192149038827143 | 0.0586614426748725 |
| S1PR5 | -1.07584595 | 3.0818126102486 | -3.435567071 | 0.00193598970600825 | 0.0586614426748725 |
| ALDH6A1 | -1.07325708 | 2.05979682341643 | -3.420985587 | 0.00200942247673769 | 0.0595055495873703 |
| TMEM39B | 1.08795230028443 | 1.54976306762627 | 3.4103012616037 | 0.00206493448239235 | 0.0600408053240887 |
| ZNF561 | -1.096162422 | 2.02797615706058 | -3.397283421 | 0.00213457937445799 | 0.0608528464498507 |
| GSTM3 | -1.051456509 | 1.61518405646478 | -3.39682624 | 0.00213706614648857 | 0.0608528464498507 |
| CDC42EP2 | -1.306447523 | 2.34518629195256 | -3.394212139 | 0.0021513391126127 | 0.061011521923831 |
| RGS18 | 1.24589742934743 | 7.40780584317803 | 3.38283618106985 | 0.00221453315237124 | 0.0618012082597899 |
| PARL | -1.109862121 | 4.44742573361574 | -3.381948401 | 0.00221953966231522 | 0.0618012082597899 |
| IL21R | -1.009446901 | 1.77171151313122 | -3.376343132 | 0.00225140347804277 | 0.0622037249603568 |
| GRAP2 | 1.32432519218788 | 5.80145465575766 | 3.37329110636407 | 0.00226893863794574 | 0.0623667235866112 |
| ARHGAP6 | 1.17576100029882 | 2.36881521700693 | 3.35235803502596 | 0.00239281640119716 | 0.0649392197995787 |
| PRCC | -1.043453803 | 3.68817739852469 | -3.337258528 | 0.00248620354813534 | 0.0660566269452734 |
| SMIM3 | 1.14328474555557 | 2.89272258526118 | 3.33117102896695 | 0.00252484068392758 | 0.0663389513031953 |
| HBA1 | -1.846551201 | 8.55819311564958 | -3.32326252 | 0.00257590227009563 | 0.0666996916314616 |
| KPTN | -1.407557081 | 1.3282518750639 | -3.322306016 | 0.00258214513267756 | 0.0667002308971167 |
| IFI30 | -1.06806549 | 6.32093936478059 | -3.317837191 | 0.00261150602746388 | 0.0670613210509602 |
| ZBTB17 | -1.196904508 | 2.32019711542802 | -3.302907547 | 0.00271195039175864 | 0.0680109383568509 |
| BRI3 | -1.308264925 | 4.36706874905057 | -3.300726375 | 0.00272693344721856 | 0.0681411398574256 |
| SBK1 | -1.084362548 | 2.0785815067329 | -3.299806919 | 0.0027332733338333 | 0.0681411398574256 |
| ZNF703 | -1.19202598 | 2.12109830833769 | -3.298333531 | 0.00274346236684703 | 0.0682364653656616 |
| CCL4L2 | -1.83587512 | 2.92253771401487 | -3.291384314 | 0.00279201460382192 | 0.0686476985159886 |
| C5AR1 | -1.226781952 | 3.59972173094253 | -3.271471845 | 0.00293576818500698 | 0.0697862917601184 |
| TWISTNB | -1.095175879 | 2.58137583492417 | -3.271119148 | 0.00293837758667931 | 0.0697862917601184 |
| TMEM106C | 1.10392427879007 | 4.25944151174104 | 3.26896873705377 | 0.00295433561888354 | 0.0697862917601184 |
| C6orf89 | -1.041214093 | 2.61812054110162 | -3.267039481 | 0.00296872349837199 | 0.0699444305550499 |
| DNMBP | -1.007609523 | 2.06697938083828 | -3.249961863 | 0.00309906039716299 | 0.0716149271612056 |
| DDX11L2 | 1.45844037360882 | 1.40023277041573 | 3.24567480421463 | 0.00313263434701211 | 0.0722190111827309 |
| TM6SF1 | 1.18845020738097 | 2.6826194900531 | 3.24043596473377 | 0.00317413750196456 | 0.0722822476164246 |
| ABCC4 | 1.15273675232828 | 3.63712172417133 | 3.23863435444846 | 0.00318853209448316 | 0.0722822476164246 |
| LRRC8D | 1.01662183916925 | 3.3346270537267 | 3.2357864734742 | 0.00321141441112269 | 0.0724765526047057 |
| MIDN | -1.07053123 | 4.00929423383108 | -3.233252226 | 0.00323190948772034 | 0.0727858607318532 |
| SGK1 | -1.08239909 | 2.46962547296166 | -3.210400335 | 0.00342248760873776 | 0.0749814256107291 |
| CD38 | -1.149951595 | 1.72669004356016 | -3.20739901 | 0.003448306233777 | 0.0749814256107291 |
| LY6E | -1.058570164 | 4.59675671892171 | -3.178972974 | 0.00370229665356871 | 0.0784250000373128 |
| FAM102A | -1.157386925 | 3.38977560189154 | -3.17404425 | 0.00374812426814097 | 0.0784250000373128 |
| PLXDC2 | 1.05262098148755 | 2.41045611131318 | 3.16971442421114 | 0.00378883196694646 | 0.0784250000373128 |
| SCFD2 | 1.02039067097575 | 3.54983623887829 | 3.15847589960009 | 0.0038964817627793 | 0.0801732907811786 |
| NAT9 | -1.107653579 | 1.88083121637468 | -3.147310536 | 0.00400633145685473 | 0.0815338545058477 |
| AIMP2 | -1.129074705 | 3.57801155996233 | -3.125535928 | 0.00422915618114747 | 0.08278482094074 |
| FAM58A | -1.341948403 | 2.83572891547544 | -3.123806328 | 0.00424735555199692 | 0.08278482094074 |
| KCTD6 | -1.020106306 | 1.78817819775169 | -3.120350873 | 0.00428393968784818 | 0.0831953504596603 |
| RARA | -1.053265637 | 4.09152792888194 | -3.103905759 | 0.00446222265096281 | 0.0854992783378762 |
| KCNMB1 | 1.02963903555324 | 1.31149660290484 | 3.10281997691281 | 0.00447424020627742 | 0.0854992783378762 |
| DOCK4 | -1.287328789 | 1.92057286449098 | -3.098424505 | 0.00452320684597909 | 0.0857028675917755 |
| POU2AF1 | -1.106967027 | 2.92302280933131 | -3.098372931 | 0.00452378442237803 | 0.0857028675917755 |
| JAZF1 | -1.049619618 | 4.53348846642889 | -3.094000625 | 0.00457300721095866 | 0.0860591826857331 |
| IFIT1 | -1.04067254 | 1.92681526311302 | -3.093648645 | 0.0045769919484367 | 0.0860591826857331 |
| C6orf48 | -1.111378794 | 4.94740981428839 | -3.0902312 | 0.00461585353340962 | 0.0865069053813832 |
| TMEM126A | -1.071880591 | 3.19781750492857 | -3.086987842 | 0.00465302709123575 | 0.0867600745182799 |
| ARHGAP10 | 1.1458000593641 | 3.42783314076228 | 3.08220715773394 | 0.00470834279618769 | 0.0870683959752668 |
| USP53 | -1.178013488 | 1.98282482427398 | -3.078475897 | 0.00475195206124011 | 0.0874307953964677 |
| TMEM256 | -1.027152439 | 4.87907772507249 | -3.078227822 | 0.00475486508546088 | 0.0874307953964677 |
| MARCKSL1 | -1.136915083 | 3.9084547394785 | -3.069231338 | 0.00486166694712268 | 0.0887854679270104 |
| PTGER2 | -1.084892257 | 2.73879709763418 | -3.066537905 | 0.00489408521237787 | 0.0888236012810718 |
| TRPT1 | -1.097104096 | 2.22565641520694 | -3.050098666 | 0.00509646780011539 | 0.0903005893211741 |
| PTPRJ | 1.10397887089464 | 4.41162485265825 | 3.04464524691369 | 0.00516535111284598 | 0.0908995458150992 |
| WRB | 1.25458254101183 | 3.39616408343563 | 3.04274781746071 | 0.00518952556483371 | 0.0908995458150992 |
| CRIP2 | -1.028539025 | 3.20725832445017 | -3.040677968 | 0.00521601983269622 | 0.0908995458150992 |
| MED27 | 1.0300863669002 | 2.26647471103799 | 3.03462102661473 | 0.0052942923820394 | 0.0908995458150992 |
| LINC01089 | 1.29708504180028 | 2.38603091295993 | 3.03349888163524 | 0.0053089160665732 | 0.0908995458150992 |
| ATP2A3 | 1.05516366869955 | 4.00162439340349 | 3.03211928523342 | 0.00532694761957443 | 0.0908995458150992 |
| THBS1 | 1.1765126319898 | 4.31702220324947 | 3.02405536434439 | 0.00543351807536393 | 0.0909187438639215 |
| KPNA2 | -1.117079717 | 3.79000831285363 | -3.017847852 | 0.00551693577036943 | 0.0909620840364418 |
| NRGN | 1.2053843928095 | 8.35585173791543 | 3.01285970081668 | 0.00558485027192683 | 0.0911389644932989 |
| ADRB2 | -1.009331906 | 1.77117457380974 | -3.009935042 | 0.00562503957129255 | 0.0911389644932989 |
| HIST1H2AC | 1.05091529990107 | 6.78893788884706 | 2.99149600224694 | 0.0058848326970663 | 0.0918408731986637 |
| PTGES | -1.06737271 | 0.727419711484732 | -2.989114672 | 0.00591920499766254 | 0.0918408731986637 |
| TFB1M | -1.087928604 | 1.92576154294896 | -2.988499346 | 0.00592811767031579 | 0.0918408731986637 |
| NAF1 | -1.133832465 | 2.06111176970388 | -2.9884141 | 0.00592935341632726 | 0.0918408731986637 |
| NFKBIE | -1.091216592 | 2.89088480366971 | -2.986110131 | 0.00596284536662622 | 0.0921061993231024 |
| CERS4 | -1.027151497 | 1.49988717793833 | -2.974549037 | 0.00613363950475973 | 0.0922602298779603 |
| MTX1 | -1.118344902 | 1.68947095222958 | -2.972242868 | 0.00616826093940562 | 0.0922602298779603 |
| ITGA2B | 1.65799045475379 | 5.2566986010425 | 2.9704789121281 | 0.0061948677096108 | 0.0923176643462667 |
| FCER1A | -1.203294863 | 1.25786217358181 | -2.96865529 | 0.00622248912598932 | 0.0923613482717417 |
| SIAE | 1.17881758438602 | 2.17015663509997 | 2.96538600490623 | 0.00627230051696156 | 0.0923613482717417 |
| ARG2 | 1.0522499105466 | 2.1950392868986 | 2.96206621860237 | 0.00632326898520805 | 0.0928567719471648 |
| TREML1 | 1.2349505851778 | 4.68640430788614 | 2.95343670447662 | 0.00645760471809631 | 0.093674590768596 |
| FAM216A | 1.08874410658945 | 2.50647567280941 | 2.93912185700882 | 0.00668643977510961 | 0.0954257574172771 |
| SIGMAR1 | -1.135988736 | 2.22510522483068 | -2.938133361 | 0.0067025226114315 | 0.0954257574172771 |
| TEC | 1.12040043160351 | 2.17940088914206 | 2.93334169006954 | 0.00678100481104942 | 0.0957080027896169 |
| ZNF485 | -1.119057895 | 1.35789563687557 | -2.93198005 | 0.00680346555724074 | 0.0957080027896169 |
| RUFY1 | 1.04697720482643 | 5.92539637887114 | 2.9209712611291 | 0.00698766921603813 | 0.0968647455541072 |
| CLCF1 | -1.129824347 | 1.08518520873602 | -2.914025405 | 0.00710631292726408 | 0.0980433392281479 |
| OSBPL5 | -1.076683555 | 2.61143216388227 | -2.906962056 | 0.00722892018478939 | 0.0984676294548187 |
| SPIN2B | 1.16838681899126 | 2.13758007213528 | 2.87804290540369 | 0.00775211557361787 | 0.101716865298878 |
| ACTN1 | 1.06813561447357 | 5.30725200240531 | 2.87571511724096 | 0.00779575208696421 | 0.101791062572785 |
| RPL23AP7 | 1.60278942258682 | 3.12654909333777 | 2.85853533500508 | 0.00812506124542188 | 0.103930377038687 |
| CCL3L3 | -1.148343964 | 1.28635905288305 | -2.84985043 | 0.00829649889004396 | 0.104584489717125 |
| PTGS1 | 1.21009098664788 | 4.83296539265077 | 2.84538146830045 | 0.00838604103211439 | 0.104993685396847 |
| INTS1 | -1.003672413 | 2.29815897938243 | -2.837679605 | 0.008542504440164 | 0.105366913984646 |
| TPTEP1 | 1.09150928086895 | 3.2345312664209 | 2.83738669889669 | 0.00854850889043673 | 0.105366913984646 |
| TNFSF12 | -1.033660252 | 3.55258642582213 | -2.829939844 | 0.00870251103802458 | 0.105928737333763 |
| TNFRSF4 | -1.056464891 | 1.39686361722039 | -2.828929062 | 0.00872361486149778 | 0.106028516230449 |
| TCP11L2 | -1.153209858 | 4.32544142134399 | -2.822624215 | 0.00885634567971242 | 0.106495980366808 |
| CCL4 | -1.278537486 | 4.66533529837065 | -2.811934086 | 0.00908575860577928 | 0.107386253863235 |
| LRRC41 | -1.033342358 | 1.99791724129344 | -2.809937884 | 0.00912921365927505 | 0.107618847966347 |
| YIF1B | 1.21768719985304 | 2.39318472821921 | 2.80664720487561 | 0.00920127592781248 | 0.108036887126122 |
| CCT6P1 | 1.1865639304803 | 3.27914486684209 | 2.79426088072933 | 0.00947735693446264 | 0.109537089252468 |
| MIPEP | -1.102366396 | 1.62193722312629 | -2.784957962 | 0.009689821115798 | 0.109920510435296 |
| PPP1R14A | 1.04371829305736 | 4.19225799242327 | 2.76489728190098 | 0.0101633244208734 | 0.112436365110178 |
| HES1 | -1.046400011 | 2.42386052485122 | -2.708766328 | 0.0116063402320297 | 0.119525073061049 |
| ZNF185 | 1.16892504802212 | 3.92794580345549 | 2.6906644493732 | 0.0121112433576053 | 0.121140606527291 |
| COPZ2 | -1.255520845 | 1.65033171594293 | -2.679911727 | 0.012420818415099 | 0.121813713229147 |
| TPPP3 | -1.293600136 | 2.2146961420931 | -2.671661012 | 0.0126633607131537 | 0.12229840256307 |
| PLAUR | -1.155144041 | 3.4914608007446 | -2.667211956 | 0.0127959776686638 | 0.122954574107047 |
| HLA-DRB5 | -1.53224867 | 1.51187923411574 | -2.662824028 | 0.012928042757132 | 0.123739837818264 |
| PSMB5 | -1.058633499 | 3.49934329397165 | -2.654890157 | 0.0131700691382577 | 0.124081915148835 |
| C16orf74 | 1.1013560116205 | 1.40579951524463 | 2.65277500282447 | 0.013235303546288 | 0.124333862341889 |
| IL1RN | -1.225892796 | 2.31553172183702 | -2.634722867 | 0.0138044554142513 | 0.126266008567213 |
| TAZ | -1.072848334 | 1.94102049491459 | -2.629118808 | 0.0139857325428837 | 0.127164591059977 |
| ACYP1 | 1.08402103929202 | 1.89349713235447 | 2.62576884648235 | 0.0140951524341463 | 0.127726148853802 |
| CDC14C | 1.08016641968006 | 1.69193672979754 | 2.61651002759303 | 0.0144017366327293 | 0.128870297748629 |
| CD151 | 1.05500736182631 | 3.50440348204209 | 2.59241570020408 | 0.0152289108429489 | 0.131206369886257 |
| SYS1 | -1.017923453 | 2.06524051781031 | -2.586501872 | 0.015438582628043 | 0.131978951971787 |
| MERTK | -1.005456443 | 1.54945291552045 | -2.577831651 | 0.0157508349117239 | 0.133057523643583 |
| PANX1 | 1.02376215477584 | 2.90295139527177 | 2.54179853367291 | 0.0171124101195873 | 0.139078875270642 |
| CLIC3 | -1.015503032 | 4.14859323948573 | -2.537621945 | 0.0172770974571722 | 0.139148542879092 |
| TYMP | -1.120372467 | 5.14500577944323 | -2.523191864 | 0.0178574530506965 | 0.140840338425572 |
| IAH1 | -1.061144311 | 2.26104326569946 | -2.485177115 | 0.0194737629118091 | 0.145652514016064 |
| CCL3 | -1.343984364 | 1.96727328966553 | -2.463819812 | 0.020440030853103 | 0.148352830565514 |
| XCL1 | -1.0253247 | 1.6308600232033 | -2.445315691 | 0.0213127167582209 | 0.149948992129112 |
| CXCR3 | -1.052814328 | 1.36025019214418 | -2.443476526 | 0.0214013020482781 | 0.149948992129112 |
| ALPK3 | -1.035053693 | 0.908056634111291 | -2.407309167 | 0.0232135613555738 | 0.155407646831597 |
| WHAMMP3 | 1.06703718673015 | 3.3657930424089 | 2.40192744567679 | 0.0234949506809257 | 0.155664938998469 |
| IFI6 | -1.154427896 | 3.51518851942522 | -2.400735263 | 0.0235577067761843 | 0.155791867144168 |
| HMOX1 | -1.091405831 | 4.82573372191777 | -2.390747247 | 0.0240895374434738 | 0.157559390722415 |
| IFI27 | -1.062739968 | 3.2600780945872 | -2.36341964 | 0.0256013105252514 | 0.162314960263281 |
| IL15RA | -1.046578373 | 2.3368054853743 | -2.251208171 | 0.0327590812550599 | 0.182051503916144 |
| NUDT14 | -1.015887032 | 1.69183605229941 | -2.238677338 | 0.0336618227832741 | 0.183828191664136 |
| IRF7 | -1.002624892 | 3.3331281443474 | -2.233995054 | 0.0340048688077771 | 0.184768220892851 |
| ZBED5-AS1 | 1.0054907455339 | 3.51964912603862 | 2.21957511340047 | 0.0350812561393667 | 0.188140087094278 |
| CFD | -1.09232112 | 3.10837404050807 | -2.190536614 | 0.0373425305118846 | 0.193149369658889 |
| CBR3 | -1.102351512 | 1.78746955374975 | -2.164725236 | 0.0394616029942745 | 0.196692961760215 |
| LINC00892 | 1.10090933517295 | 2.3621868133435 | 2.15613550256659 | 0.0401903602705968 | 0.196910723080803 |
| HLA-C | 1.04980558097845 | 6.57273170452374 | 2.14842099578898 | 0.0408551141324046 | 0.19790638206027 |
| FAM185A | 1.14878427085267 | 2.09806166041237 | 2.12195358989175 | 0.0432112032767079 | 0.202644714890177 |
| PRR7 | 1.20874093391998 | 2.59900963652189 | 2.11745810313357 | 0.0436232229400411 | 0.203338865210392 |
| CXCL2 | -1.071654515 | 1.97890568005085 | -2.103280356 | 0.044945685986084 | 0.206677743401466 |
